# Cell-type-specific analysis of alternative polyadenylation using single-cell transcriptomics data

**DOI:** 10.1093/nar/gkz781

**Published:** 2019-09-10

**Authors:** Eldad David Shulman, Ran Elkon

**Affiliations:** Department of Human Molecular Genetics and Biochemistry, Sackler School of Medicine, Tel Aviv University, Tel Aviv, Israel

## Abstract

Alternative polyadenylation (APA) is emerging as an important layer of gene regulation because the majority of mammalian protein-coding genes contain multiple polyadenylation (pA) sites in their 3′ UTR. By alteration of 3′ UTR length, APA can considerably affect post-transcriptional gene regulation. Yet, our understanding of APA remains rudimentary. Novel single-cell RNA sequencing (scRNA-seq) techniques allow molecular characterization of different cell types to an unprecedented degree. Notably, the most popular scRNA-seq protocols specifically sequence the 3′ end of transcripts. Building on this property, we implemented a method for analysing patterns of APA regulation from such data. Analyzing multiple datasets from diverse tissues, we identified widespread modulation of APA in different cell types resulting in global 3′ UTR shortening/lengthening and enhanced cleavage at intronic pA sites. Our results provide a proof-of-concept demonstration that the huge volume of scRNA-seq data that accumulates in the public domain offers a unique resource for the exploration of APA based on a very broad collection of cell types and biological conditions.

## INTRODUCTION

The maturation of mRNA 3′ ends is a two-step process, termed *cleavage and polyadenylation*, that involves endonucleolytic cleavage of the nascent RNA followed by synthesis of a poly(A) tail at the 3′ terminus of the cleaved product (1). Cleavage and polyadenylation sites (pA sites) are defined by adjacent RNA sequence *cis*-elements, with a key role involving the AAUAAA signal (called the *polyadenylation signal* (*PAS*)), typically located ∼20 nt upstream of the pA site. There are >10 weaker variants of this canonical PAS, the main one being AUUAAA (2). Auxiliary elements include upstream U-rich and UGUA motifs and downstream U-rich and GU-rich elements. The strength of a pA site is determined by these elements in a combinatorial manner (3).

Over the last decade, several deep-sequencing techniques were developed for the precise mapping of the 3′ ends of transcripts (4). Importantly, these transcriptome-wide methods revealed that the majority of human protein-coding genes contain more than one 3′ untranslated region (3′ UTR) pA site, indicating *alternative polyadenylation* (*APA*) as a widespread regulatory layer that generates transcript isoforms with alternative 3′ ends (1,5,6). APA in the 3′ UTR typically generates mRNA isoforms with markedly different 3′ UTR lengths. For example, it was observed that for mouse, the median 3′ UTR lengths of shortest and longest APA isoforms differ ∼7-fold, at 250 nt and 1770 nt, respectively (1,6). As 3′ UTRs contain *cis*-elements that serve as major docking platforms for microRNAs (miRNAs) and RNA binding proteins (RBPs), which are involved in various aspects of mRNA metabolism, 3′ UTR APA can potentially affect post-transcriptional regulation in multiple ways, including the modulation of mRNA stability, translation efficiency, nuclear export and cellular localization (4,7,8). Yet, our current understanding of the impact of APA on gene regulation is still very rudimentary. Somewhat unexpectedly, two recent studies failed to detect a large effect of 3′ UTR shortening on transcript stability or translation efficiency (9,10).

Transcriptomic studies demonstrated that APA is globally modulated in response to changes in cell proliferation and differentiation. Global 3′ UTR shortening was first documented during the activation of T cells, B cells and monocytes (11). Further analysis of a large panel of diverse human tissues and cell lines revealed a significant anti-correlation between proliferation and 3′ UTR length indices, establishing the universality of the link between the proliferative state of cells and APA. This association between proliferation status and 3′ UTR length extends to cancer cells, which typically express shorter 3′ UTRs compared with non-transformed cells (12,13). Notably, it was indicated that switching to shorter 3′ UTRs allows proto-oncogenes (e.g., *CCND1*) to avoid inhibition by miRNAs, thereby enhancing their tumorigenic activity (12). Additionally, analysis of various cellular models showed that differentiation is accompanied by 3′ UTR lengthening; in contrast, the generation of induced pluripotent stem cells (iPSCs) from differentiated cells was accompanied by global 3′ UTR shortening (14,15). We previously showed that the expression of many factors of the polyadenylation machinery is elevated in proliferative cells (16), providing one potential mechanism for the enhanced usage of proximal pA sites in proliferation. Yet, depletion of factors of the polyadenylation machinery does not always result in global 3′ UTR lengthening, arguing for more complex effects for individual factors, where some promote the use of proximal pA sites while others enhance the usage of distal ones (17). Furthermore, we observed that in addition to enhanced usage of proximal 3′ UTR pA sites, highly proliferative cells also show enhanced premature cleavage and polyadenylation at intronic pA sites (16). Several factors protect cells against such premature cleavage at cryptic pA sites within introns, as the usage of these sites may generate non-functional transcripts (18–20). Recently, pervasive intronic polyadenylation was detected in immune cells which diversifies their proteome by C-terminal domain loss (21).

Novel single-cell RNA sequencing (scRNA-seq) technologies allow the definition and molecular characterization of different cell types to an unprecedented degree, and their usage is growing at an exponential pace (22,23). Notably, some of the most popular scRNA-seq protocols, often called 3′ tag-based methods, generate sequence reads that are enriched at the 3′ ends of transcripts (e.g. InDrop (24), CEL-seq2 (25), Drop-seq (26), MARS-seq (27) and SCRB-seq (28)). scRNA-seq analyses have already led to profound discoveries of novel cell types and lineage differentiation trajectories (23,29,30). scRNA-seq was recently used to study patterns of stochastic gene expression (31) and cell-to-cell variability in pA site selection within homogeneous cell populations (32). We reasoned that scRNA-seq data generated by 3′ tag-based methods could also be utilized for systematic examination of APA modulation between different cell types. Analysing diverse scRNA-seq datasets that profiled very different tissues and biological processes, we demonstrate here that this rich resource allows global exploration of cell-type-specific APA regulation.

## MATERIALS AND METHODS

A detailed description of the bioinformatics pipeline that we implemented for APA analysis based on 3′ tag scRNA-seq data is provided in Supplementary Methods.

The code and sample input files for this pipeline are available at https://github.com/ElkonLab/scAPA.

### Definition of 3′ UTR regions

We used GENCODE (33) annotations to define sets of disjoint 3′ UTR regions for human and mouse genes (GENCODE releases v27 and M18, respectively). As a gene can have transcripts with different 3′ UTRs and different annotated transcripts often have the same 3′ UTR, we defined, per gene, a set of non-overlapping 3′ UTRs (Supplementary Figure S1A). This processing defined a set of 27 151 3′ UTR regions of 23 204 human protein-coding genes and a set of 25 780 3′ UTRs of 19 940 mouse protein-coding genes. In those cases where a 3′ UTR region spans two exons (1183 and 8351 such cases in human and mouse genes, respectively), we considered only the region of the last exon as the 3′ UTR.

### Processing raw scRNA-seq data

Summary statistics for the four scRNA-seq datasets analysed in our study are provided in Supplementary Table S1. For the brain dataset (SRA accession: SRP135960), we downloaded the publicly available aligned BAM files. In the other datasets, for which aligned files were not available (mouse T cells (GSE106264), sperm cells (GSE104556) and lung tumour (ArrayExpress, accession E-MTAB-6149)), we downloaded the fastq files and aligned the reads using Cell Ranger 2.1.0 *cellranger count* with default parameters (24), using hg19 and mm10 references. PCR duplicates were removed using the UMI-tools *dedup* procedure (34). As the UMI-tool dedup requires that each line in the BAM file has a molecular barcode tag, we first filtered the BAMs, leaving only reads with a corrected molecular barcode tag, using Drop-seq tools version 1.13 *FilterBAM* (TAG_RETAIN = UB) (26). We next merged all reads that originated from cells assigned to the same cell cluster into a single BAM file, based on cell assignments to clusters as provided by the original publications of the datasets and using the Drop-seq tool *FilterBAMByTag* and the SAMtools *merge* utility (35). In each dataset, this processing generated one BAM file for each cell cluster.

### Peak identification and quantification

Peaks were identified using Homer *findPeaks* (36) (using size = 50, minDist = 1) and using bedtools *merge* (37) to merge overlapping peaks. Only uniquely mapped reads were used for peak identification. Peaks within 3′ UTRs were identified using intersection (bedtools *intersect*) with the 3′ UTR annotation files described above. A peak that intersected a 3′ UTR got an ID composed of the 3′ UTR’s ID and a sequential peak index, starting with the most proximal peak (5′-most location) (Supplementary Figure S1B). Adjacent pA sites may result in a single peak. We used mclust Gaussian finite mixture models (38) to identify such events and split them into two peaks (Supplementary Figure S1C). Specifically, for each peak, we fit a bimodal model and sought those that showed clear separation between the two components. Empirically, we observed that requiring that the two modes be separated by more than three standard deviations of the fitted Gaussians and by at least 75 nt worked well for this task (Supplementary Figure S1C). On a training set that we built based on visual inspection of the peaks and which included 158 unimodal and 42 bimodal cases, the above criterion showed a sensitivity of 81% (34 out of 42 cases) with no false positive splits (specificity of 100%). In the four datasets, we analysed in this study, the proportion of peaks that were split by this step was 8–11%.

Once the genomic intervals of the peaks were determined, the number of reads that mapped to each peak in each cell-cluster BAM file was counted using the featureCounts function from the Rsubread package (39).

### Peak filtering and benchmarking

First, in each dataset, after normalizing peak counts to units of counts per million (CPMs), we considered only peaks that had more than a total sum of 10 CPMs over all the cell clusters. Next, as the usage of oligo-dT primers by scRNA-seq protocols can yield reads that originate from priming internal A-rich regions within the transcript (rather than from the transcript's 3′ end poly(A) tail), to avoid false calls of pA sites we filtered out peaks with downstream A-rich sequences. Specifically, peaks having a genomic sequence of at least eight consecutive As in the region between 10 nt to 140 nt downstream of their 3′ edge were suspected to result from internal priming and were excluded from the analysis.

Peaks were analysed for enriched motifs using DREME (40). The 3′ UTR peaks were benchmarked against annotated human and mouse pA sites from PolyA DB (release 3.1) (41). (We used UCSC liftOver (42) to convert the coordinates of the mouse annotated pA sites from mm9 to mm10 assembly.) The distance between each peak's 3′ edge and its nearest annotated pA site was calculated using bedtools *closest*, requiring the same strand (-s).

### Tests for differential APA patterns between cell clusters

3′ UTRs that showed differential usage of pA sites between different cell types were detected using chi-squared tests, performed on peak counts and corrected for multiple comparisons using BH FDR correction. 3′ UTRs with a q-value below 0.05 were considered significant. To quantify the relative usage of the most proximal pA site within a 3′ UTR (with two or more peaks), we defined the proximal pA site usage index (*proximal PUI*). For a given 3′ UTR, the proximal PUI is defined by:

$$\begin{equation*}{\rm proximal}\ {\rm PUI}\ = {\log _2}\left( {\frac{{{C_1} + 1}}{{\left\langle {C + 1} \right\rangle }}} \right)\ ,\end{equation*}$$

where ${C_1}$ is the read count of the proximal peak, and $\langle C \rangle$is the geometric mean of the counts of all the peaks associated with the 3′ UTR. To avoid zeroes in the denominator and in the log function, we added a pseudo count of 1 to all before calculating the PUI. We compared proximal PUI distributions of different cell clusters using either Wilcoxon's or Kruskal–Wallis tests, when two or more than two cell types were compared, respectively.

We considered 3′ UTRs with exactly two peaks to identify events of 3′ UTR shortening (or lengthening). (In all analysed datasets, typically 80% of the 3′ UTRs that showed a dynamic change in APA had exactly two peaks.) For such 3′ UTRs, an increase in the value of the proximal PUI in cell type 1 relative to cell type 2 indicates 3′ UTR shortening in cell type 1. The global tendency for 3′ UTR shortening or lengthening in one cell cluster relative to another was tested using a one-tailed binomial test.

For 3′ UTRs with more than two peaks that showed a significant differential usage of pA sites, the usage of each peak was tested separately (using chi-squared tests).

All tests were performed in R-3.4.4 and plots were made using ggplot2 R package.

### Analysis of intronic pA sites

Analysis of pA sites in introns was performed in a similar manner to 3′ UTR analysis. We used the bedtools *intersect* procedure to intersect the peaks’ bed file with a bed file of intronic regions downloaded from the UCSC table browser (43), using the track of GENCODE release v27 (as used for the 3′ UTR analysis). We filtered out intronic regions that intersected 3′ UTRs. We then used featureCounts to create an intron count matrix, similar to the matrix created for 3′ UTRs. We filtered out intronic peaks with less than a total of 50 counts and 10 CPMs over all the cell clusters. We further filtered out intronic peaks with a genomic sequence of seven consecutive As in the region from 1 nt to 200 nt downstream of the peak's 3′ edge.

To identify changes in the relative usage of intronic versus 3′ UTR pA sites, we compared the counts of each intronic peak to the sum of the counts of the 3′ UTRs that are of the same gene and are downstream of the intronic peak. Per intronic pA site, differential relative usage was identified using chi-squared tests (with FDR of 5%). Per intronic pA site and cell cluster, we calculated the intronic pA site usage index:

$$\begin{equation*}{\rm intronic}\ {\rm PUI}\ = {\log _2}\left( {\frac{{{C_i} + 1}}{{{C_U} + 1}}} \right)\ ,\end{equation*}$$

where ${C_i}$ is the count of reads mapped to the intronic peak, and ${C_U}$is the sum of the counts of the reads mapped to all the 3′ UTRs of that gene in that cell cluster. Comparing different cell types, higher intronic PUI indicates elevated usage of the intronic pA site.

### Expression analysis

Starting with the filtered 3′ UTR peaks count matrix, we summed the count of all peaks in each 3′ UTR to obtain a count matrix with UTR IDs as rows and cell clusters as columns. We then normalized this matrix by transforming counts to CPMs followed by quantile normalization.

## RESULTS

### Analysis of APA modulation in activated T cells

**Figure 1.**
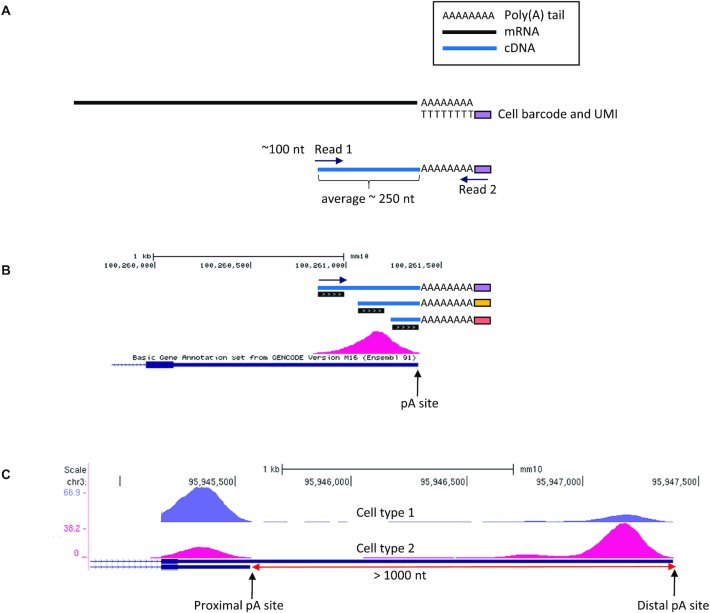
Utilizing 3′ tag scRNA-seq data for the study of APA. (**A**) Key steps in library preparation of 3′ tag-based scRNA-seq methods. Oligo-dT primers anneal to the poly(A) tail and ligate the cell barcode and unique molecular identifier (UMI) to the RNA molecules. After various preparation steps including fragmentation and amplification, short cDNA fragments that contain the poly(A) tail are selected for sequencing. Sequenced reads are generated from the opposite end of the fragment (Read1) in addition to their paired-end shorter mates that sequence the barcodes (Read2). (**B**) Reads originating from different RNA molecules of the same transcript isoform pile up to a peak whose edge is at or near the pA site. Shorter fragments, such as the one with orange barcode, yield reads closer to the pA site. Fragments of medium size (such as the fragment with the yellow barcode) are more common, and thus the centre of the peak has a higher density of reads. (**C**) scRNA-seq data from different cell types allow identification of changes in the relative usage of alternative pA sites.

The 3′ tag-based scRNA-seq methods use oligo-dT primers, which anneal to the poly(A) tail of transcripts for ligating the cell barcode to the RNA molecules. Library preparation of these protocols generates short cDNA fragments (typically ∼200–300 bp) that contain the cell barcodes and the start of the poly(A) tail at one of their ends. Sequenced reads (of the typical length of ∼100 nt) are generated from the opposite end of the fragment (Figure 1A) in addition to their paired-end shorter mates that sequence the barcodes. Because the fragmentation processes implemented in these protocols are stochastic, different RNA molecules of the same transcript isoform result in fragments of different lengths. Reads derived from shorter fragments end closer to the pA site, while reads from longer fragments end further from the pA site. Therefore, such scRNA-seq protocols generate aligned reads that pile up to form peaks at genomic intervals adjacent to pA sites (Figure 1B). Considering collectively all the individual cells that belong to a certain cell type, accumulation of the reads that originate from a common pA site forms a peak that allows the quantification of the usage of the pA site in that cell type. Comparisons between different cell types enable exploration of APA modulation and identification of events of significant change in the relative usage of alternative pA sites within a 3′ UTR (Figure 1C).

**Figure 2.**
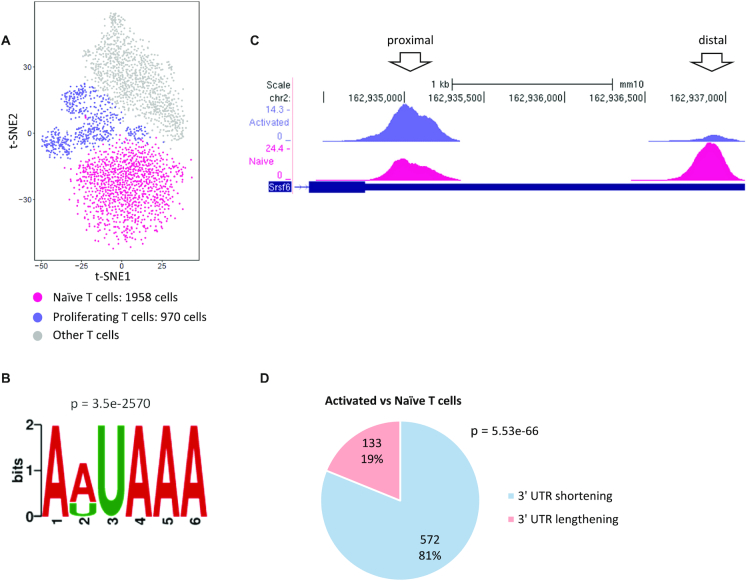
Analysis of APA modulation in activated T cells. (**A**) t-SNE plot of all the T cells from wild type mice identified by Pace *et al.* (44). We analysed the clusters of proliferating T cells (970 cells; purple) and naïve T cells (1,978 cells; pink), defined in the original publication. (**B**) The top-scoring signal detected by *de novo* motif analysis of the 9611 3′ UTR peaks corresponds to the canonical PAS motif and its main variant. (Sequences used for this analysis spanned the region from 30 nt upstream to 120 nt downstream of the peaks 3′ edge.) (**C**) An example of a gene that shows significant 3′ UTR shortening in activated T cells (top track) compared with naïve T cells (bottom track). (**D**) A pie chart for the distribution of 3′ UTRs with exactly two peaks that showed a significant change in pA site usage in the comparison between proliferating and naïve T cells. (*P*-value calculated using single-tailed binomial test.)

Global elevation of usage of proximal pA sites, which results in widespread 3′ UTR shortening, was first observed in activated T cells (11), and therefore as a first test case, we analysed a scRNA-seq dataset that profiled *in vivo* murine T cells (44). Standard scRNA-seq analysis of this dataset defined the main T cell subpopulations (Figure 2A). Here, we focused on the naïve and activated T cell populations (containing 1958 and 970 cells, respectively). Merging all uniquely mapped 3′ UTR reads in the dataset, we identified 3′ UTR peaks and quantified their usage in each cell-type cluster (see Materials and Methods and Supplementary Figure S1). After filtering out peaks with overall low usage and those suspected to stem from internal priming (Materials and Methods and Supplementary Figure S2A), a total of 9611 3′ UTR peaks were detected in this dataset. Reassuringly, *de novo* motif analysis demonstrated that the genomic sequences around these peaks were significantly enriched for the canonical PAS motif (AAUAAA) and its main variant (AUUAAA), which together were detected in 79% of the peaks (Figure 2B and Supplementary Figure S2B). As expected, the PAS signal was located close to the 3′ end of the peaks (Supplementary Figure S2C) and peaks with higher reads coverage had a greater chance to span the PAS signal (Supplementary Figure S2D). This result is corroborated by benchmarking the 3′ edge of these peaks against their closest annotated pA sites from the PolyA DB (41) (Supplementary Figure S2E).

Next, we searched for dynamic APA events, that is, cases that showed a significant change in the relative usage of alternative 3′ UTR pA sites between naïve and activated T cells (Figure 2C). In total, 868 such events were identified in this dataset (FDR = 5%). Notably, in line with previous reports, these events were strongly inclined towards 3′ UTR shortening in the proliferating cells compared with the resting ones (Figure 2D and Supplementary Figure S2F). Thus, these results provided a proof-of-principle finding for the ability to mine 3′ tag scRNA-seq data for exploration of APA regulation.

### Modulation of the 3′ UTR length of transcripts during sperm cell differentiation

**Figure 3.**
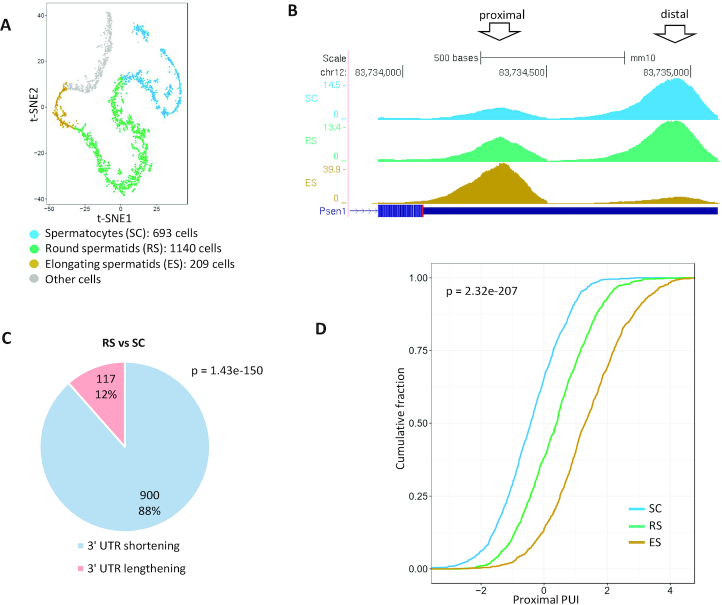
Analysis of APA modulation during spermatogenesis. (**A**) scRNA-seq defines different cell types during spermatogenesis in mice (48). Clustering analysis separated cells in different developmental stages (t-SNE coordinates and cell assignment to clusters are from the original publication). (**B**) An example of a gene that shows gradual 3′ UTR shortening in the transition from spermatocytes (top blue track) to elongating spermatids (bottom golden track). (**C**) APA analysis detected significant 3′ UTR shortening in 900 genes in the comparison between RS and SC cells (*P*-value calculated using single-tailed binomial test). (Similarly strong results were obtained for the comparison between ES and RS cells (Supplementary Figure S3D)). (**D**) Distribution of the proximal PUI index of transcripts showing significant change in pA site usage in SC, RS and ES cells (p-value calculated using Kruskal–Wallis test). The gradual shift to the right reflects the dynamics of this phenomenon.

Bulk experiments found that sperm cell differentiation is among the biological processes that show the most drastic alterations in APA patterns and widespread 3′ UTR shortening (45–47). Therefore, we next analysed a scRNA-seq dataset that examined this process (48). While bulk analysis of the testis inevitably merges sperm cells from different maturation stages into one pool, analysis based on scRNA-seq allows refined separation of the cells into distinct sets according to developmental stage. In the analysis of the mouse sperm cell scRNA-seq dataset, we focused on three stages along this differentiation process: (i) early-stage—spermatocytes; (ii) intermediate-stage—round spermatids and (iii) late-stage—elongating spermatids (Figure 3A). Applying the same method for identifying and filtering peaks that we used above for the T cell dataset, 10 506 3′ UTR peaks were detected in this dataset. These peaks too were significantly enriched for the PAS signals (Supplementary Figure S3A–C). Tests for differential usage of alternative 3′ UTR pA sites revealed a very sharp and progressive increase in the relative usage of proximal 3′ UTR sites, detected for 900 transcripts in the comparison between round spermatids and spermatocytes and for >880 transcripts in the comparison between elongating spermatids and round spermatids (Figure 3B and C; Supplementary Figure S3D). In addition, comparison of the distribution of the *proximal PUI* indexes (which quantify the relative usage of the proximal 3′ UTR pA sites (Methods)) over the three stages demonstrated the gradual evolvement of the immense 3′ UTR shortening during the progression through the sperm cell maturation trajectory (Figure 3D).

### Analysis of APA patterns of the brain

**Figure 4.**
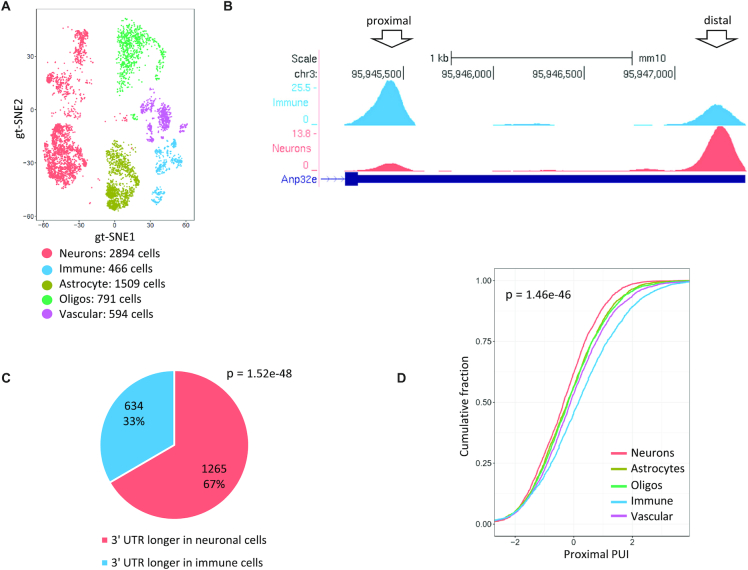
Analysis of APA modulation in brain cells. (**A**) scRNA-seq dataset defined different cell types in the mouse cortex and dorsal midbrain (52). Shown is gt-SNE (a modification of t-SNE). Coordinates and cell assignment into clusters are as provided by the original publication. (**B**) An example of a gene whose 3′ UTR is markedly longer in neuronal cells compared with immune cells in the brain. (**C**) Global APA analysis showed significant 3′ UTR lengthening in neurons compared with immune cells in the brain (*P*-value calculated using single-tailed binomial test). (**D**) Cumulative distributions of the proximal PUI index of all the cell types identified in the brain sample. Note that the curves of the neurons and immune cells are, respectively, the most left and right ones, reflecting the most pronounced enhancement and attenuation of 3′ UTR proximal pA site usage in these cell types (*P*-value calculated using Kruskal–Wallis test).

APA analyses of bulk tissues demonstrated that APA patterns are tissue-specific (5,49), suggesting that APA contributes to tissue-specific gene regulation (50). In this context, the brain has been shown to preferentially use distal pA sites to a large degree (51). To dissect cell-type-specific APA patterns within a complex tissue, we analysed a scRNA-seq dataset from mouse cortex and dorsal midbrain (52). Five main cell types were delineated in this dataset: neurons, astrocytes, immune cells, oligodendrocytes and vascular cells (Figure 4A). In total, we detected in this dataset 16 942 3′ UTR peaks, which showed enrichment for the expected PAS signal at the expected location upstream of the putative pA sites (Supplementary Figure S4A–C). Interestingly, 2506 transcripts showed a significant change in pA site usage across these cell types, and the most pronounced difference was between neuronal cells and brain immune cells, which manifested the strongest preference for longer and shorter transcripts, respectively (Figure 4B–D). While most brain cells have their embryological origin in the ectoderm, brain immune cells originate from the mesoderm. Our results are in line with the finding that in *Drosophila melanogaster*, the preference for long isoforms in the brain begins in the ectoderm (53).

### APA modulation in lung cancer

**Figure 5.**
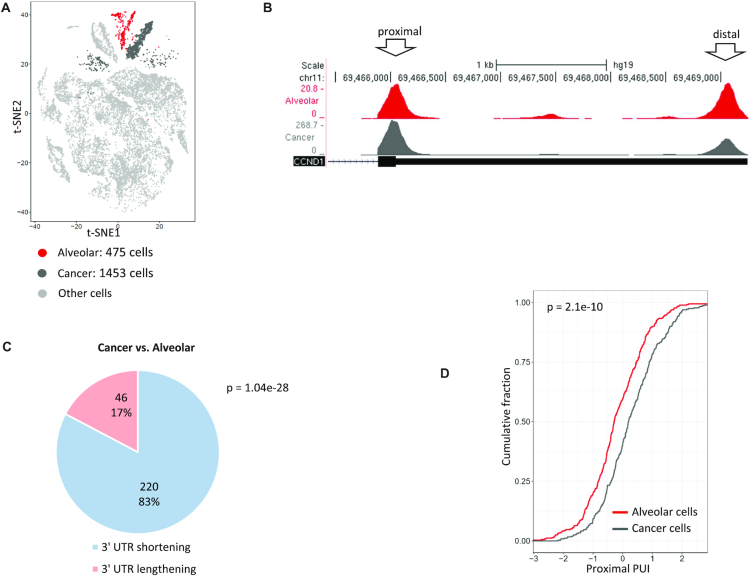
Analysis of 3′ UTR APA in lung tumour sample. (**A**) t-SNE plot of the cells detected in a lung tumour sample (57). t-SNE coordinates and cell assignment into clusters are as provided in the original publication. (**B**) An example of a gene, the proto-oncogene *CCND1*, that shows significant 3′ UTR shortening in the cancer cells compared with their normal counterparts. (Similar effect was observed for hundreds of genes.) Of note, previous reports demonstrated that enhanced usage of the proximal pA site of *CCND1* augments its oncogenic activity by generating shorter and more stable transcripts (12). (**C**) A pie chart of the distribution of transcripts that show a significant change in pA site usage between cancer and normal alveolar cells within the lung sample. In pink and blue are the proportions of transcripts with shortened or lengthened 3′ UTRs in the cancer cells, respectively (*P*-value calculated using single-tailed binomial test). (**D**) Cumulative distribution of the proximal PUI index in alveolar cells (red) and cancer cells (dark grey) (*P*-value calculated using single-tailed Wilcoxon test).

Studies in cell lines and tumour tissues indicate that malignant transformation is accompanied by global 3′ UTR shortening (12,54). Furthermore, these studies suggest that identification of shortened transcripts may improve the prediction of patients’ prognoses (55,56). These previous APA analyses were carried out on bulk tumour tissues. The single-cell analysis allows a much finer view of programmes of APA modulation that occur in the transformed cells, by enabling direct comparison between cancer cells within a solid tumour and their normal counterpart cells. To this end, we next analysed scRNA-seq data from a sample taken from a non-small cell lung cancer patient (57). scRNA-seq dissected this heterogeneous sample into its main constituent cell types and indicated that in this tumour, the cancer cells originated from alveolar epithelial cells (Figure 5A). Analysing 1453 cancer cells and 475 alveolar cells, we detected overall 9542 3′ UTR peaks in this dataset that were also enriched for the PAS signals (Supplementary Figure S5A–C). In line with previous reports, cancer cells showed a significant 3′ UTR shortening that occurred in dozens of transcripts (Figure 5B–D). We next examined if 3′ UTR shortening in cancer cells correlates with their proliferative state. Interestingly, using the expression of *PCNA* and *CCND1*, two hallmark cell-cycle genes, as a proxy for proliferation status, we did not observe a significant difference between high- and low-proliferating cancer cells (Supplementary Figure S5D).

**Figure 6.**
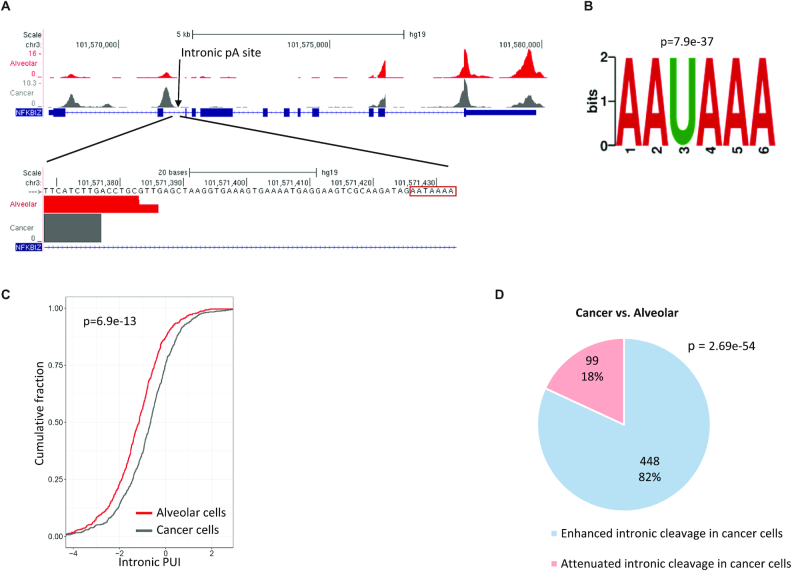
Analysis of intronic APA in lung tumour sample. (**A**) An example of a gene that shows, in the comparison between cancer and normal cells, a significant increase in the usage of an intronic pA site. This intronic site harbours the canonical AAUAAA signal at the expected location. (Note that in addition to the enhanced cleavage at the intronic pA site, this gene also shows the typical increased usage of the 3′ UTR proximal pA site in cancer cells compared with the normal ones.) (**B**) The PAS signal was the top-scoring motif detected by *de novo* motif analysis applied to the 3057 intronic peaks that were left in the analysis after the removal of putative internal priming peaks. (**C**) Cumulative distribution of the *Intronic PUI index* in alveolar cells (red), and cancer cells (dark grey) (*P*-value calculated using single-tailed Wilcoxon test). The shift to the right of the cancer cells’ curve reflects enhanced cleavage at intronic pA sites. (**D**) A pie chart illustrating the distribution of intronic pA sites that showed a significant change in their usage in the comparison between cancer and alveolar cells. In blue and pink, respectively, are the proportions of intronic pA sites with enhanced or attenuated usage in cancer cells (*P*-value calculated using single-tailed binomial test).

We and others previously observed that 3′ UTR shortening that results from enhanced cleavage at proximal pA sites is often accompanied by augmented cleavage at cryptic pA sites within introns (16,19). Therefore, we expanded the analysis of this lung cancer dataset and examined patterns of aberrant intronic polyadenylation in the cancer cells compared with those of their normal counterparts. We initially detected 8249 intronic peaks with a total of >50 reads and 10 CPMs. However, examination of the genomic sequences downstream of these peaks revealed a very significant enrichment for the A-rich motif (Supplementary Figure S6A), which was much stronger here compared with the prevalence of this motif in peaks detected in 3′ UTRs (a prevalence of ∼55% in intronic peaks compared with <15% in 3′ UTR ones; compare Supplementary Figure S6A with Supplementary Figures S2A, S3A, S4A and S5A). This result indicates that a large portion of the peaks identified within introns originated from internal priming to A-rich regions within primary RNA molecules rather than from genuine polyadenylation sites. To reduce this noise of false calls, we thus applied a stricter filtering criterion and excluded any intronic peak with a sequence of seven or more consecutive As in the genomic region from 1 nt to 200 nt downstream of their 3′ end. Reassuringly, the top-scoring enriched motif in the remaining 3057 intronic peaks was the canonical PAS motif (Figure 6A–B and Supplementary Figure S6B).

We next searched for differences in the relative usage of intronic and 3′ UTR pA sites between cancer and alveolar cells. Overall, we detected 547 significant changes. To determine the direction of each change, we defined per gene the *intronic pA site usage index* (*Intronic PUI*) in analogy to the *proximal PUI* defined above (Methods), where a higher value of this index reflects higher usage of the intronic site relative to the 3′ UTR sites. Cancer cells showed a significant increase in this index compared with the normal alveolar cells (Figure 6C). Remarkably, 82% of the differential events showed elevated usage of the intronic pA site in the cancer cells (Figure 6D).

### Robustness of APA analysis using 3′ tag scRNA-seq data

To examine the robustness of APA analysis based on scRNA-seq data generated by 3′ tag methods, we tested the effect of reads coverage on the number of identified 3′ UTR pA sites. Random downsampling analysis showed that typical sequencing depth used in current datasets allows robust detection of 3′ UTR pA sites which is nearing saturation. For example, reducing the number of reads to as low as 20% of the original amount still detected 95% (∼10 150 out of 10 500 and ∼16 600 out of 17 000 in the full spermatogenesis and brain cells datasets, respectively) of the 3′ UTR peaks identified (Supplementary Figure S7A–B). Detection of events of differential usage of pA sites between cell types (dynamic APA events) was more sensitive to reads coverage, but still showed good robustness: for example, reducing the number of reads to as low as 20% of the original amount decreased the number of detected APA events to ∼67% (∼800 out of 1200 events) of the number of events detected in the full spermatogenesis dataset (Supplementary Figure S7C) and to ∼50% (∼1200 out of 2500 events) in the brain cells dataset (Supplementary Figure S7D).

In recent years, several bioinformatics tools were developed to infer APA modulation from bulk RNA-seq datasets, including DaPars (Dynamic analyses of Alternative PolyAdenylation from RNA-Seq) (13) and Change-Point (58). Similar to the pipeline, we implemented in this study, both DaPars and Change-Point aim to identify 3′ UTR APA switching events without relying on prior annotations of pA sites. We next examined how these tools perform on 3′ tag scRNA-seq data, using the T-cells dataset as a test case. On this dataset, our peak-detection-based approach identified 868 events of 3′ UTR APA modulation, with a strong preference for 3′ UTR shortening in activated cells (Figure 2). While DaPars detected only a small number of APA switching events (<40 events), Change-Point called >1100 dynamic events, which also showed global 3′ UTR shortening in activated T cells (Supplementary Figure S8A). While the overlap between the dynamic events detected by the peak approach and Change-Point is significant (369 events), the majority of events were detected by only one of the methods (Supplementary Figure S8B). The peaks approach showed a clear advantage in delineating the location of the pA sites (Supplementary Figure S8C) and accordingly showed much more significant enrichment of the PAS motif (Supplementary Figure S8D). Inspection of dynamic events called by only one of these methods, showed that peaks called only by Change-Point often involved weak pA sites (that did not have enough reads coverage to be called by the peak detection procedure) (Supplementary Figure S8E), while those called only by the peak approach often involved 3′ UTR peaks that spanned into the gene coding region (Supplementary Figure S8F). To enhance the identification of APA dynamic events, the pipeline we implemented for APA analysis using scRNA-seq data, allows the user to choose either the peak detection method or the Change-Point method.

**Figure 7.**
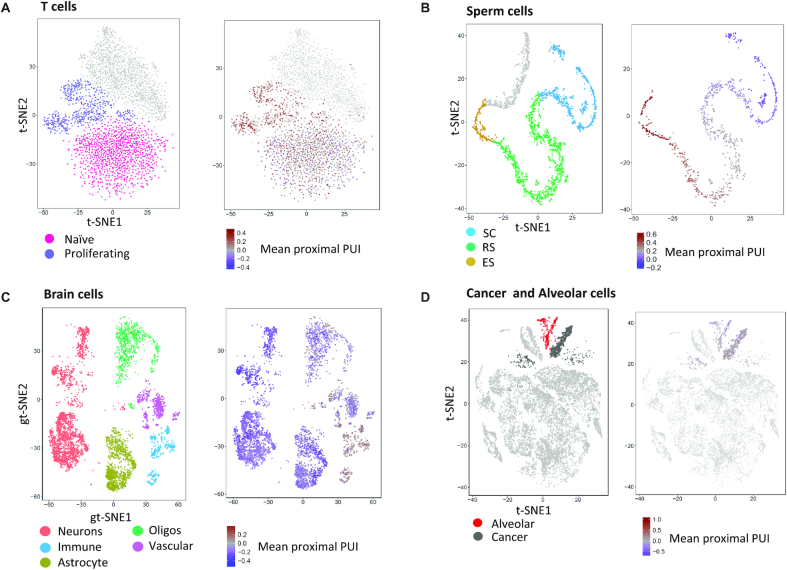
Single-cell-level exploration of APA. For each cell, we calculated the mean proximal PUI index over all the 3′ UTRs with more than one peak covered by at least one read. Despite limited coverage, APA status strongly correlated with cell type also at the level of individual cells.

Last, while our analysis builds on single-cell data, given the limited coverage of individual cells (in the analysed datasets, the average number of reads in 3′ UTR peaks per cell is 8000–14 000), we did not perform single-cell-level analysis but rather pooled reads across all the cells that were assigned to the same cluster (that is, those that belong to the same ‘cell type’). Nevertheless, we sought to examine whether, despite limited coverage, current single-cell data can discern single-cell-level APA modulation. For this task, we calculated, per cell, the mean proximal PUI, where higher levels reflect elevated pA activity resulting in enhanced cleavage at proximal pA sites (and thus 3′ UTR shortening). Interestingly, in all the datasets, this analysis delineated the main APA patterns at the level of individual cells (Figure 7).

## DISCUSSION

In this study, we provide a strong demonstration for the utility of scRNA-seq data generated by 3′ tag-based methods for the analysis of APA, despite it not being intentionally developed for the study of this regulatory layer. By analysing single-cell (SC) data, from T cells we detected the global 3′ UTR shortening that is associated with the proliferative state, and by analyzing SC data from spermatogenesis we delineated the drastic 3′ UTR shortening that accompanies this developmental trajectory. The analysis of SC data from the brain pinpointed neurons as the cell type that is characterized as having the greatest incidence of longer isoforms, whereas the analysis of a lung tumour showed global aberration of APA in cancer cells, manifested by enhanced cleavage at both proximal 3′ UTR and intronic pA sites.

By comparison of different cell types or different biological conditions, analysis of 3′ tag-based transcriptomic data globally delineates changes in the relative expression of short versus long gene isoforms. Such changes can stem in principle from either differential activity of the APA machinery that alters the balance between usage of proximal and distal pA sites or from the differential activity of factors that regulate mRNA stability (e.g., miRNAs, RBPs). Only experimental analysis can reveal the underlying causal mechanism (that is, differential APA or stability). Furthermore, the observed changes probably reflect the combined effect of these two regulation modes. Accordingly, it was shown that in spermatogenesis, during the differentiation of spermatocytes into spermatids, the relative expression of the isoforms with shorter 3′ UTRs is drastically elevated compared with the longer ones due to both increased cleavage at proximal 3′ UTR pA sites and enhanced mRNA degradation of the longer isoforms that contain destabilizing elements (46,47,59–61).

The functional impact of APA on gene expression is largely unknown. Since miRNAs mainly have a repressive effect on target genes, 3′ UTR shortening is expected to be generally associated with increased stability. In contrast to this expectation, recent studies observed that global 3′ UTR shortening in mouse fibroblasts and T cells is not accompanied by significant changes in mRNA or protein levels (9,10). In other biological conditions, we previously did observe a link between enhanced cleavage at proximal pA sites that led to 3′ UTR shortening and elevated expression levels (19). In three of the four scRNA-seq datasets analysed here, we too observed a significant association between 3′ UTR shortening and increased expression (Supplementary Figure S9), supporting mainly destabilizing roles for 3′ UTR regulatory elements. Yet, it emerges that the impact of 3′ UTR APA on miRNA-mediated regulation of mRNAs is more complicated than mere inclusion/exclusion of miRNA target sites in/from the 3′ UTR, as the efficiency of mRNA targeting is also affected by the location of the miRNA target site—sites located at the start or end of the 3′ UTRs are more efficient than those located in the middle (62). Thus, APA can modulate the activity of a miRNA target site by changing its location relative to the transcript's 3′ end (63). Taking this effect into account, a recent study estimated that APA influences ∼10% of miRNA targeting between any two cell types compared (62).

In the analysis of the lung cancer dataset, we observed that the cancer cells showed increased cleavage at both proximal 3′ UTR and intronic pA sites. These results are in line with a recent study that implicated premature cleavage and polyadenylation at intronic pA sites, indicating it as yet another process that is harnessed by cancer cells for selective proliferative advantage (64). It emerges, therefore, that aberrant APA can enhance cancer transformation either by the stabilization of proto-oncogenes through shortening of their 3′ UTRs or by the inactivation of tumour suppressor genes (TSGs) through premature cleavage at cryptic pA sites within their introns. However, in the dataset we analysed, while many cancer genes (based on COSMIC gene annotations (65)) were affected by APA modulation in cancer cells (20 oncogenes and 10 TSGs; Supplementary Table S2), we did not observe an enrichment for oncogenes among those showing 3′ UTR shortening and nor did we see enrichment for TSGs among those showing enhanced intronic cleavage. The rapid accumulation of scRNA-seq data from various cancer types will allow wide-scale evaluation of how general these functional APA events are in the process of tumorigenesis. In line with our observations, very recently Ye *et al.* analysed 3′ tag scRNA-seq data to explore the dynamics of APA in acute myeloid leukaemia (AML) and observed elevated APA modulation in AML patients compared with healthy controls (66).

An inherent limitation of APA analysis using 3′ tag scRNA-seq data is the resolution of pA site detection. The characteristic width of the peaks hampers the separation between very close pA sites. Using pA site annotations from PolyA DB, we found that in the analysed datasets, while we robustly separated adjacent pA sites with distances above 300–400 nt, we were able to separate ∼30% of successive sites whose distance is 200–300 nt and only ∼5% of the sites whose distance is <200 nt (Supplementary Figure S10).

APA is still a largely unexplored layer of gene regulation. The study of APA was hampered for years by the lack of widely adopted transcriptomic techniques for quantification of pA usage that allowed systematic analysis of APA modulation. Our analyses demonstrate that the huge volume of 3′-tag scRNA-seq data that accumulates in the public domain fills this gap, and provides a unique resource for exploration of APA under a very broad collection of cell types and biological conditions. It thus holds great promise for improving our understanding of the roles of APA in normal physiological processes and the development of pathological conditions.

## Supplementary Material

gkz781_Supplemental_File
