# Supplementary material for: Cell-type-specific analysis of alternative polyadenylation using single-cell transcriptomics data
Source: Nucleic Acids Res. 2019 Sep 10;47(19):10027–39. doi: 10.1093/nar/gkz781 (PMC6821429; doi:10.1093/nar/gkz781)
Supplement: gkz781_Supplemental_File [file gkz781_supplemental_file.pdf]

Supplementary Table 1

Dataset 1: T cells (GSE106264)

**# Peaks**	9,634
**# Genes with peaks**	7,681

	**Total (KO and WT)**	**Activated (WT)**	**Naive (WT)**
**# Mapped reads reads**	265,491,250	28888825	29,908,646
**# Uniquely mapped reads**	185,698,999	21239038	23,198,822
**# Cells**	8,585	970	1,958

Dataset 2: Spermatogenesis (GSE104556)

**# Peaks**	10,524
**# Genes with peaks**	8,213

	**Total**	**SC**	**RS**	**ES**	**CS**	**Sertoli**	**Spg**	**Leydig**
**# Mapped reads**	143,038,126	55,168,233	61,688,975	11,794,866	13,525,157	289,343	426,518	145,034
**# Uniquely mapped reads**	114,967,127	43,609,005	49,059,602	10,014,156	11,747,440	219,941	201,696	115,287
**# Cells**	2,552	693	1,140	209	492	7	6	5

Dataset 3: Brain cells (SRP135960)

**# Peaks**	17,082
**# Genes with peaks**	12,350

	**Total**	**Astrocytes**	**Ependymal**	**Immune**	**Neurons**	**Oligos**	**Vascular**
**# Mapped reads**	189,242,685	19,142,128	1,965,831	5,729,951	120,605,726	26,417,082	15,381,967
**# Uniquely mapped reads**	182,945,094	18,611,268	1,893,788	5,484,538	116,492,057	25,695,117	14,768,326
**# Cells**	6,337	1,509	83	466	2,894	791	594

Dataset 4: Lung Cancer (ArrayExpress, E-MTAB-6149)

**# Peaks**	9,373
**# Genes with peaks**	7,536

	**Total (Pt. 3,4,5 Cancer and Alveolar cells)**	**Alveolar sample 3**	**Cancer sample 3**
**# Mapped reads**	76,476,424	23,580,205	17,040,360
**# Uniquely mapped reads**	64,825,943	20,314,154	14,120,376
**# Cells**	4,972	1,453	475

Supplementary Table 2

a. Cancer genes with significant 3' UTR APA modulation in the lung cancer dataset

Gene Symbol	Ensembl Gene ID	Role in Cancer	Shortening/Lengthening in Cancer	p-Value	q-Value
BIRC3	ENSG00000023445	Oncogene, Tumor Suppressor	Shortening	1.69E-11	2.07E-08
CCND1	ENSG00000110092	Oncogene	Shortening	4.52E-52	6.05E-49
ELF3	ENSG00000163435	Tumor Suppressor	Shortening	9.23E-15	1.16E-11
ETNK1	ENSG00000139163	Tumor Suppressor	Shortening	4.11E-12	5.05E-09
EWSR1	ENSG00000182944	Oncogene	Shortening	1.33E-07	1.5E-4
H3F3A	ENSG00000163041	Oncogene	Lengthening	1.17E-25	1.53E-22
H3F3B	ENSG00000132475	Oncogene	Shortening	4.64E-10	5.55E-07
HNRNPA2B1	ENSG00000122566	Oncogene	Shortening	1.12E-33	1.48E-30
JUN	ENSG00000177606	Oncogene	Shortening	7.37E-09	8.59E-06
LARP4B	ENSG00000107929	Tumor Suppressor	Shortening	6.18E-06	6.3E-03
MAPK1	ENSG00000100030	Oncogene	Shortening	4.74E-08	5.41E-05
MAX	ENSG00000125952	Tumor Suppressor	Lengthening	1.64E-05	1.6E-02
PTEN	ENSG00000171862	Tumor Suppressor	Shortening	4.17E-05	4.0E-02
SRSF2	ENSG00000161547	Oncogene	Lengthening	1.54E-18	1.97E-15
STAT3	ENSG00000168610	Oncogene	Shortening	2.68E-15	3.37E-12
TPM3	ENSG00000143549	Tumor Suppressor	Shortening	1.06E-09	1.26E-06
WWTR1	ENSG00000018408	Oncogene	Shortening	4.18E-09	4.90E-06

b. Cancer genes with significant intronic APA modulation in the lung cancer dataset

Gene Symbol	Ensembl Gene ID	Role in Cancer	Enhanced/Attenuated Cleavage in Cancer	p-Value	q-Value
BAZ1A	ENSG00000198604	Tumor Suppressor	Enhanced	9.74E-06	2.4E-02
BRD3	ENSG00000169925	Oncogene	Enhanced	1.50E-06	3.9E-03
BRD4	ENSG00000141867	Oncogene	Enhanced	1.37E-14	3.91E-11
CCND1	ENSG00000110092	Oncogene	Attenuated	9.21E-33	2.70E-29
DEK	ENSG00000124795	Oncogene	Enhanced	1.89E-8	5.09E-05
ETNK1	ENSG00000139163	Tumor Suppressor	Attenuated	1.24E-8	3.34E-05
HNRNPA2B1	ENSG00000122566	Oncogene	Enhanced	7.05E-26	2.06E-22
KDM5A	ENSG00000073614	Oncogene	Enhanced	3.13E-06	7.9E-04
KLF6	ENSG00000067082	Tumor Suppressor	Enhanced	6.23E-22	1.81E-18
MET	ENSG00000105976	Oncogene	Attenuated	6.97E-16	2.00E-12
NPM1	ENSG00000181163	Oncogene	Enhanced	7.39E-09	2.01E-05
NR4A3	ENSG00000119508	Oncogene	Enhanced	4.30E-29	1.28E-25
RAC1	ENSG00000136238	Oncogene	Enhanced	2.61E-07	6.8E-04
ROS1	ENSG00000047936	Oncogene	Attenuated	5.11E-13	1.44E-09
RPL22	ENSG00000116251	Tumor Suppressor	Enhanced	1.67E-126	4.96E-123
RUNX1	ENSG00000159216	Oncogene, Tumor Suppressor	Enhanced	1.57E-16	4.52E-13

Supplementary Methods

Detailed description of our pipeline for APA analysis from 3' tag scRNA-seq data

Scripts implementing the pipeline and input sample files are available from https://github.com/ElkonLab/scAPA

Our pipeline consists of the following 5 steps:

1. Defining 3'UTR peaks
2. Quantifying the usage of each peak in each cell cluster
3. Filtering peaks
4. Detecting dynamical APA events
5. Inferring global trends of APA modulation

Input Files:

- Alignment BAM files generated by cellranger count, for each of the experiment's *n* samples: $Aligned_1$.BAM $Aligned_2$.BAM … $Aligned_n$.BAM
- Cell cluster annotation files: each file contains the cell barcodes that belong to each cell cluster *j*, from sample *i* ($Cluster_{ji}$.txt).

We provide an R shell script that automatically runs all the analysis steps. The following text describes in detail the commands and tools used for the first two steps of the analysis and more general description of the other three steps ran by the pipeline.

1. Defining 3'UTR peaks

The input for this step is the alignment BAM files, generated by cellranger count, for each of the experiment's *n* samples: $Aligned_1$.BAM $Aligned_2$.BAM … $Aligned_n$.BAM

a. PCR duplicates removal

i. PCR duplicates are removed using UMI-tools *dedup*. As UMI tools dedup requires that each line in the BAM file has a molecular barcode tag, Drop-seq tools is first used to filter the BAMs, leaving only reads for which cell ranger counts produced corrected molecular barcode tag.

```
FilterBAM TAG_RETAIN=UB  I=Aligned_i.BAM O= UB.Aligned_i.BAM
```

ii. Then UMI tools is ran with "method=unique" so that cellranger corrected molecular barcodes are used:

```
umi_tools dedup -I UB.Aligned_i.BAM -S dedup.Aligned_i.BAM --method=unique --extract-umi-method=tag --umi-tag=UB --cell-tag=CB
```

b. Peak detection

i. Homer is used to create a tag directory (Tagdirectory) from the PCR duplicate removed BAMs:

```
makeTagDirectory Tagdirectory dedup.Aligned1.BAM dedup.Aligned2.BAM …
```

ii. Homer findPeaks is used to identify peaks. By default, findPeaks adjusts reads to the center of their fragment. To avoid this, fragLength is set to the average read length. In order to find peaks of variable width, Homer is set to find peaks of width 50nt and a minimum distance of 1 nt between peaks.

```
findPeaks Tagdirectory -size 50 -fragLength 100 -minDist 1 -strand separate -o Peakfile
```

iii. Bedtools is used to merge peaks less than 100 nt apart

```
mergeBed -d 100 -s –i  peakfile > merge.peakfile
```

iv. Intersect peaks file with a 3' UTR bed file to create a GTF of the 3' UTR peaks:

```
bedtools intersect -wa -wb -s -a merge.peakfile -b 3UTR.BED
```

The output file is edited to produce a valid bed file (peaks.BED) where the peaks are annotated according to their 3' UTR and their position within it.

c. Separating Peaks with bimodal UMI counts distribution

Adjacent pA sites may result in a single peak. To detect and separate such peaks the R package *mclust* is used to fit a Gaussian finite mixture model with two components to the UMI counts distribution in the interval of each peak. The input to mclust, the UMI counts distribution, is prepared as follows:

i. To detect reads from the union of all reads from all samples, the duplicate-removed BAM files are merged.

```
samtools merge merged. Aligned.BAM  dedup.Aligned1.BAM dedup.Aligned2.BAM …
```

ii. Two BEDGRAPHS files are produced from this BAM, one for the plus strand and one for the minus strand, using bedtools genomcove

```
bedtools genomcov -strand +(-) -bg -ibam merged. Aligned.BAM  > covrage.plus(minus).wig
```

iii. The BEDGRAPHS files are converted into a BED format and bedtools intersect is used to intersect them with the peaks' BED file.

iv. The intersected file is read in R and converted to a list such that each element of the list, corresponding to a specific peak, is a numeric vector whose values represent read coverage observed across the peak.

v. mclust is used to fit an equal variance Gaussian finite mixture model with two components to (G=2, modelNames="E") to each list element.

vi. If the predicted means of the two fitted Gaussian components are separated by more than three standard deviations and at least 75 nt, the peak is split into two, according to mclust classification.

vii. The peak's bed is edited accordingly (Correct peak index).

2. Quantifying the usage of each peak in each cell cluster

This step uses *featureCounts* to count the reads that overlap each peak in each cluster ("cell type").

i. A separate BAM file for each cell cluster is generated. First, Drop-seq tools is used to split the reads in each sample BAM into separate BAMs that correspond to the different clusters. This is done using *FilterBAMByTag* together with text files ($Cluster_{ji}$.txt), where each file contains the cell barcodes that belong to each cell cluster *j*, from sample *i*.

```
FilterBAMByTag TAG=CB TAG_VALUES_FILE= Clusterji.txt I= dedup.Alignedi.BAM
O= Clusterji.BAM
```

ii. Next, for each cluster *j* all *n* files corresponding to this cluster are merged to produce one BAM file for that cluster ($Cluster_j$ .BAM):

```
samtools merge Clusterj .BAM Clusterj1.BAM Clusterj2.BAM Clusterjn.BAM …
```

iii. Rsubread package *featureCounts* function is used, where the annotation file is a SAF data.frame edited from the peaks bed file (peaks.SAF). largestOverlap = True is specified so that reads spanning two peaks are counted according to their largest overlap.

```
featureCounts(files = Cluster1 .BAM Cluster2 .BAM …, annot.ext = peaks.SAF,
largestOverlap = T)
```

The result is a count matrix, where the rows are peaks, and columns are cell clusters.

```
head(PeakCountMatrix)
              3' UTR ID Peak index Navie T cells Cycling T cells
ENSMUSG00000025903.14_1          1            22              91
ENSMUSG00000025903.14_2          1          1289             898
ENSMUSG00000033813.15_1          2           489             125
ENSMUSG00000033793.12_1          1           499             424
ENSMUSG00000025907.14_1          1            81              39
ENSMUSG00000025907.14_1          2            48              20
```

3. Peak filtering

a. First, in each dataset, after conversion of peak counts to counts-per-million (CPM) units, only peaks whose total sum over all cell clusters is above 10 CPMs are considered.

b. To exclude internal priming suspected peaks, peaks having a stretch of at least 8 consecutive As in the region between 10 nt to 140 nt to the peak's 3' end are filtered.

4. Statistical analysis – detection of dynamic APA events between cell clusters

a. Each 3'UTR with more than one peak is represented by a table where rows are peak indices and columns cell clusters, e.g.:

```
$ENSMUSG00000000184.12_2
                   3' UTR ID Peak index Navie T cells Cycling T cells
7243 ENSMUSG00000000184.12_2          2           871             444
7244 ENSMUSG00000000184.12_2          1          2624             763
```

For each table, we perform a Chi-squared test

b. p-values are corrected for multiple testing using BH FDR.

5. Inferring global trends of APA modulation

a. The proximal pA site usage index (*proximal PUI*) is used to quantify the relative usage of the most proximal pA site within a 3' UTR (with two or more peaks). For a given 3' UTR, the proximal PUI is defined by:

$$proximal\ PUI = \log_2\left(\frac{C_1+1}{<C+1>}\right),$$

where C_1 is the read count of the proximal peak, and $< C >$ is the geometric mean of the counts of all the peaks associated with the 3' UTR. To avoid zeros in the denominator and in the log function, a pseudo count of 1 is added to all before calculating the PUI.

b. For 3' UTR with more than two peaks that show significant usage change, for each peak *i*, chi-square test for goodness of fit is performed.

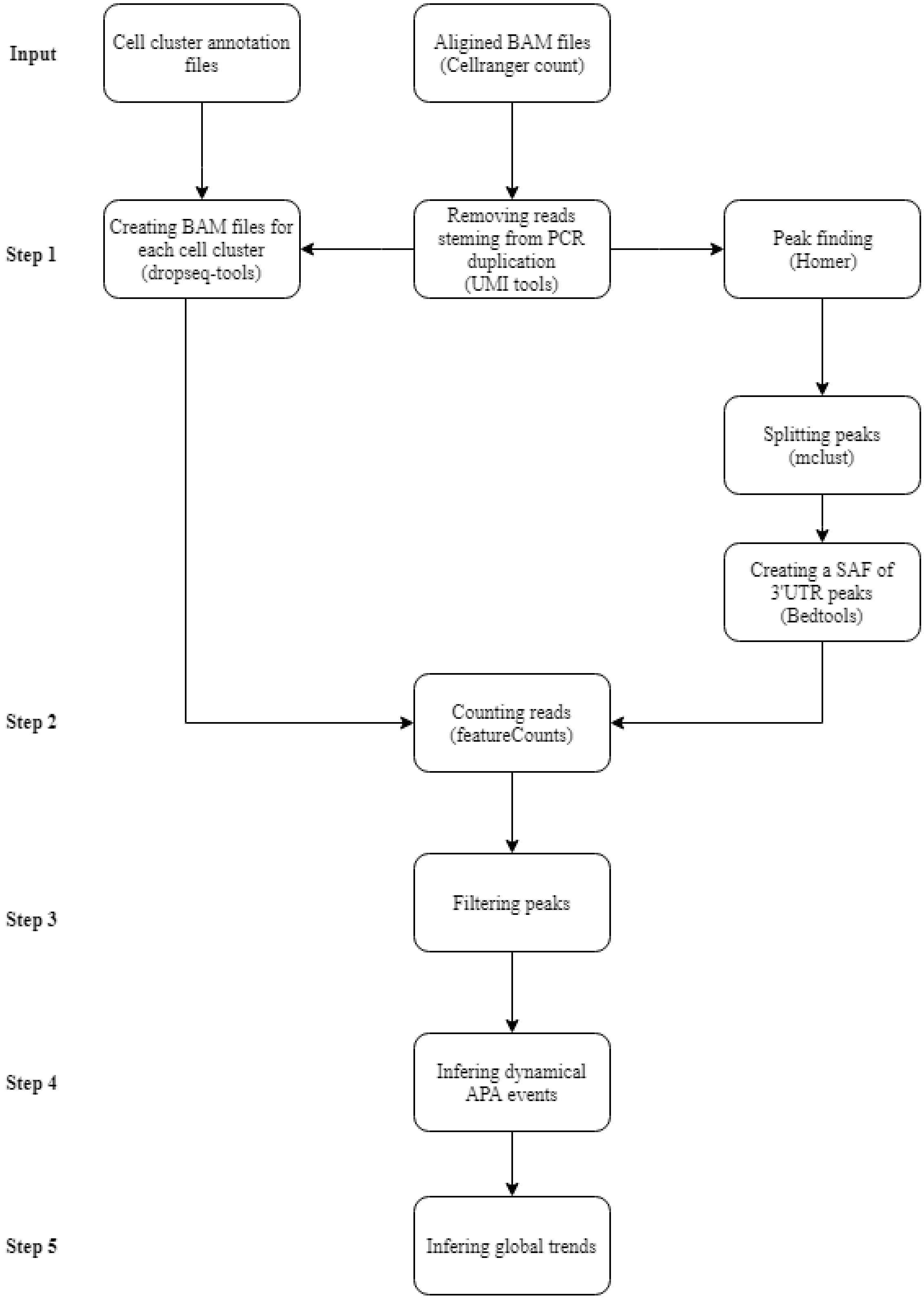
Input
Cell cluster annotation files
Aligined BAM files (Cellranger count)
Step 1
Creating BAM files for each cell cluster (dropseq-tools)
Removing reads steming from PCR duplication (UMI tools)
Peak finding (Homer)
Splitting peaks (mclust)
Creating a SAF of 3'UTR peaks (Bedtools)
Step 2
Counting reads (featureCounts)
Step 3
Filtering peaks
Step 4
Infering dynamical APA events
Step 5
Infering global trends

Supplementary Figure legends

Figure S1. (A) For the identification of 3' UTR peaks we first defined a set of disjoint 3' UTRs for human and mouse protein-coding genes. As an example, shown here are the 3' UTRs defined for the mouse *Glrx2* gene. (The numbers after the underline represent 3' UTR index, sequentially numbered from 5' to 3'.) (B) Example of the peaks detected by Homer (Methods) in the 3' UTR of the *Vezf1* gene in T cells. Peak IDs are composed of their gene ID, 3' UTR index and peak index (sequentially numbered from 5' to 3'). (C) Refinement of peaks calling. We used 2-components Gaussian mixture models (implemented by *mclust*) to identify and split two adjacent pA sites that were called by Homer as a single peak. Shown here as an example, the peak called in the 3' UTR of *Ctdsp1*. On the left is the original peak called by Homer and on the right is the two-component model fitted to the peak by mclust. The dashed vertical lines are the means of the model and the horizontal blue line represents the region that is less than 3 SDs from the first mean.

Figure S2. (A) Downstream A-rich motif was detected in ~14% (1,488/11,021) of the 3' UTR peaks (analyzed region from 1–150 nt downstream of the peaks' 3' end). These peaks were suspected to stem from internal priming and thus filtered out from the subsequent analysis. **(**B) *De novo* motif analysis of the 3' UTR peaks (that passed the two filtering steps) in the T cell dataset detected strong enrichment for the canonical PAS signal and its main variant. (analyzed region from 30 nt upstream to 120 nt downstream of the peaks' 3' end). (C) Location distribution of the PAS signals relative to the peaks' 3' end. As the PAS signal is usually located ~20 nt upstream of the pA site, its location relative to the peaks' end can be used to gauge the typical distance between a peak's end and its pA site. The mode of the location distribution of the PAS motif is at 5 nt upstream of the peak 3' edge, indicating that the identified peaks in this dataset commonly end only a few nt before the pA site. (D) Location distribution of the canonical PAS signal relative to the peaks' 3' end for peaks divided into three groups according to their read coverage. As expected, peaks with higher coverage end more closely to the pA site (and thus have the PAS closer to their ends). (E) Distribution of the distance between peaks' end and their nearest annotated pA site in PolyA DB. As a control, we also calculated such distributions for randomly selected sites in the 3' UTRs. Horizontal lines in the violin plots indicate the 25th, 50th and 75th quantiles. (F) We defined the proximal PUI (see Methods) as a measure for the usage

of the most proximal 3' UTR pA site relative to all the pA sites within the same 3' UTR. The plot compares the cumulative distribution of this index between the proliferative and naïve T cells. The shift to the right demonstrates enhanced usage of proximal pA sites in the activated state. (p-value calculated using one-tailed Wilcoxon's test).

Figure S3. (A) A-rich motif was detected downstream of ~2% (168/11,130) of the 3' UTR peaks in the spermatogenesis dataset. These peaks were suspected as stemming from internal priming and thus were filtered out from the subsequent analysis. **(**B) The remaining 3' UTR peaks were significantly enriched for the PAS signals, which were located at the expected location (and showed the expected dependence on peak coverage). (C) Distribution of the distance between peaks' end and their nearest annotated pA site in PolyA DB. (D) APA analysis detected significant 3' UTR shortening in 889 genes in the comparison between ES and RS cells (p-value calculated using single-tailed binomial test).

Figure S4. A-C. Same as **Supplementary Figure S3A-C**, but here shown for the 3' UTR peaks identified in the brain scRNA-seq dataset.

Figure S5. A-C. Same as **Supplementary Figure S3A-C**, but here shown for the 3' UTR peaks identified in the lung cancer scRNA-seq dataset. (D) Cumulative distribution of the proximal PUI index in alveolar cells (475 cells) and cancer cells (1,453 cells) divided into lowly and highly proliferative according to the expression of PCNA (left; 114 PCNA+ cancer cells) or CCND1 (right; 306 CCND1+ cancer cells).

Figure S6. (A) The peaks identified in introns in the lung tumour dataset were strongly enriched for A-rich motif, which was detected in >55% (4,696 out of 8,249) of them. Peaks with a downstream A-rich motif likely stem from internal priming rather than from genuine pA sites, and thus were filtered out from the subsequent analysis. (B) After filtering out putative internal priming peaks, the top-scoring motif corresponded to the canonical PAS (analyzed region from 20 nt upstream to 160 nt downstream of the peak's 3' end).

Figure S7. Effect of reads coverage on APA analysis. We used random downsampling to examine the effect of reads coverage on the number of detected 3' UTR pA peaks in (A) the spermatogenesis (B) and brain cell datasets, as well as on the number of dynamic APA events detected in these datasets (C and D).

Figure S8. Analysis of T cells scRNA-seq data using Change-Point. (A) Change-Point identified 1,110 events of 3' UTR APA switching, 76% of them exhibited 3' UTR shortening. (B) Overlap between 3' UTR APA switching events detected by the peaks and Change-Point methods. (C). Distribution of the distance to the closest annotated pA site of 3' ends of peaks, change-points or random sites within 3' UTRs. (D) Motif enrichment in the 3' end of the 1,927 peaks within the 868 3' UTR with significant APA events detected by the Peaks method, and in the 1,110 sites called by Change-Point, as well as the location distribution of the identified PAS signals with respect to the 3' edge of the peak (for the Peaks methods) or to the switch position detected by Change-Point. Analyzed sequences span 30 bp upstream to 120 bp downstream of the peak's 3'end/change-point. (E) Example of APA switching events detected by Change-Point but not the Peaks approach. (Horizontal line indicates the location of the change-point). (F) Example of APA switching events detected by the Peaks approach but not by Change-Point.

Figure S9. Tests for the association between change in 3' UTR length and gene expression. Each plot compares transcripts that showed significant 3' UTR shortening and lengthening. P-values calculated using Wilcoxon's test. In three comparisons (activated vs. naïve T cells, ES vs. RS sperm cells and immune cells vs. neurons), 3' UTR shortening was associated with elevated gene expression.

Figure S10. Separation of adjacent 3' UTR pA sites as a function of their distance for the brain cells (A) and spermatogenesis (B) datasets. The distance between successive pA sites was calculated from PolyA DB annotations. For each distance bin, we calculated the portion of adjacent pA pairs that were called by distinct peaks in our analysis. Only pA sites covered by at least 10 reads and detected in at least 10% of the samples in PolyA DB were included in this analysis.

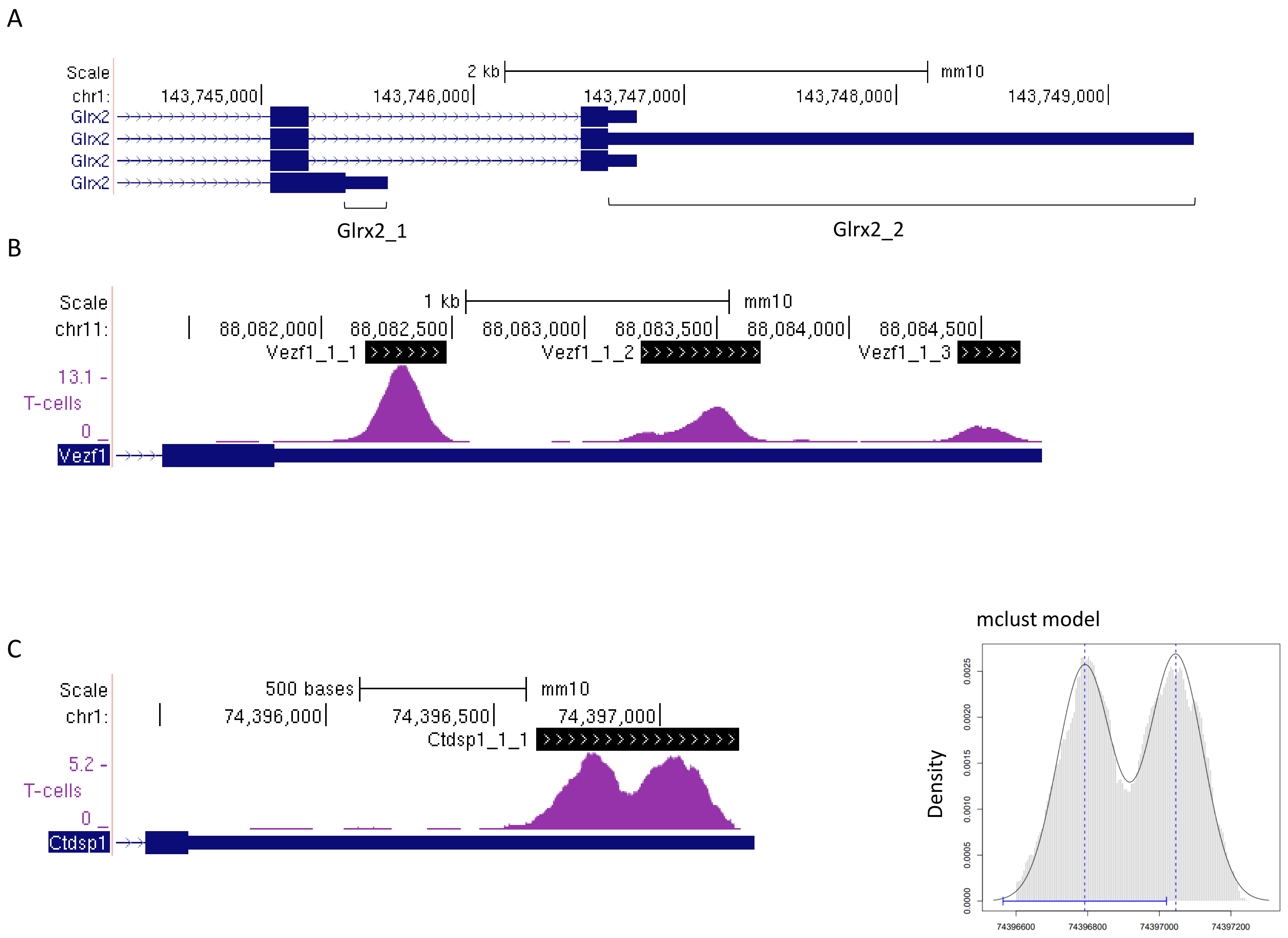

A
Scale
2 kb
mm10
chr1:
143,745,000
143,746,000
143,747,000
143,748,000
143,749,000
Glrx2
Glrx2
Glrx2
Glrx2
Glrx2_1
Glrx2_2
B
Scale
1 kb
mm10
chr11:
88,082,000
88,082,500
88,083,000
88,083,500
88,084,000
88,084,500
Vezf1_1_1
Vezf1_1_2
Vezf1_1_3
13.1 -
T-cells
0 _
Vezf1
C
Scale
500 bases
mm10
chr1:
74,396,000
74,396,500
74,397,000
Ctdsp1_1_1
5.2 -
T-cells
0 _
Ctdsp1
mclust model
Density
0.0000
0.0005
0.0010
0.0015
0.0020
0.0025
74396600
74396800
74397000
74397200

Figure S1

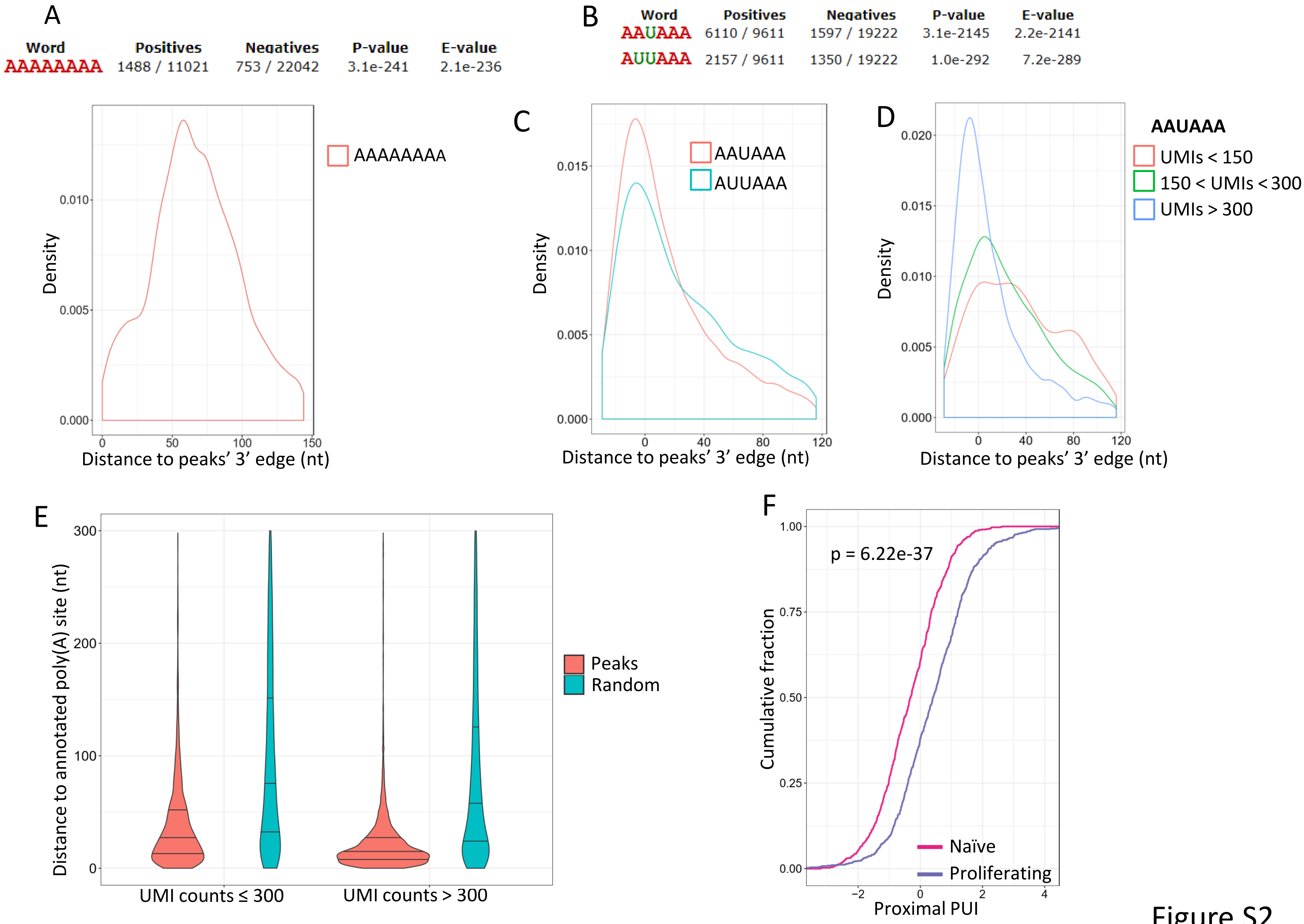
A
Word Positives Negatives P-value E-value
AAAAAAAA 1488 / 11021 753 / 22042 3.1e-241 2.1e-236
B
Word Positives Negatives P-value E-value
AAUAAA 6110 / 9611 1597 / 19222 3.1e-2145 2.2e-2141
AUUAAA 2157 / 9611 1350 / 19222 1.0e-292 7.2e-289
AAAAAAAA
Density
Distance to peaks' 3' edge (nt)
C
AAUAAA
AUUAAA
Density
Distance to peaks' 3' edge (nt)
D
AAUAAA
UMIs < 150
150 < UMIs < 300
UMIs > 300
Density
Distance to peaks' 3' edge (nt)
E
Distance to annotated poly(A) site (nt)
Peaks
Random
UMI counts ≤ 300
UMI counts > 300
F
p = 6.22e-37
Cumulative fraction
Naïve
Proliferating
Proximal PUI

Figure S2

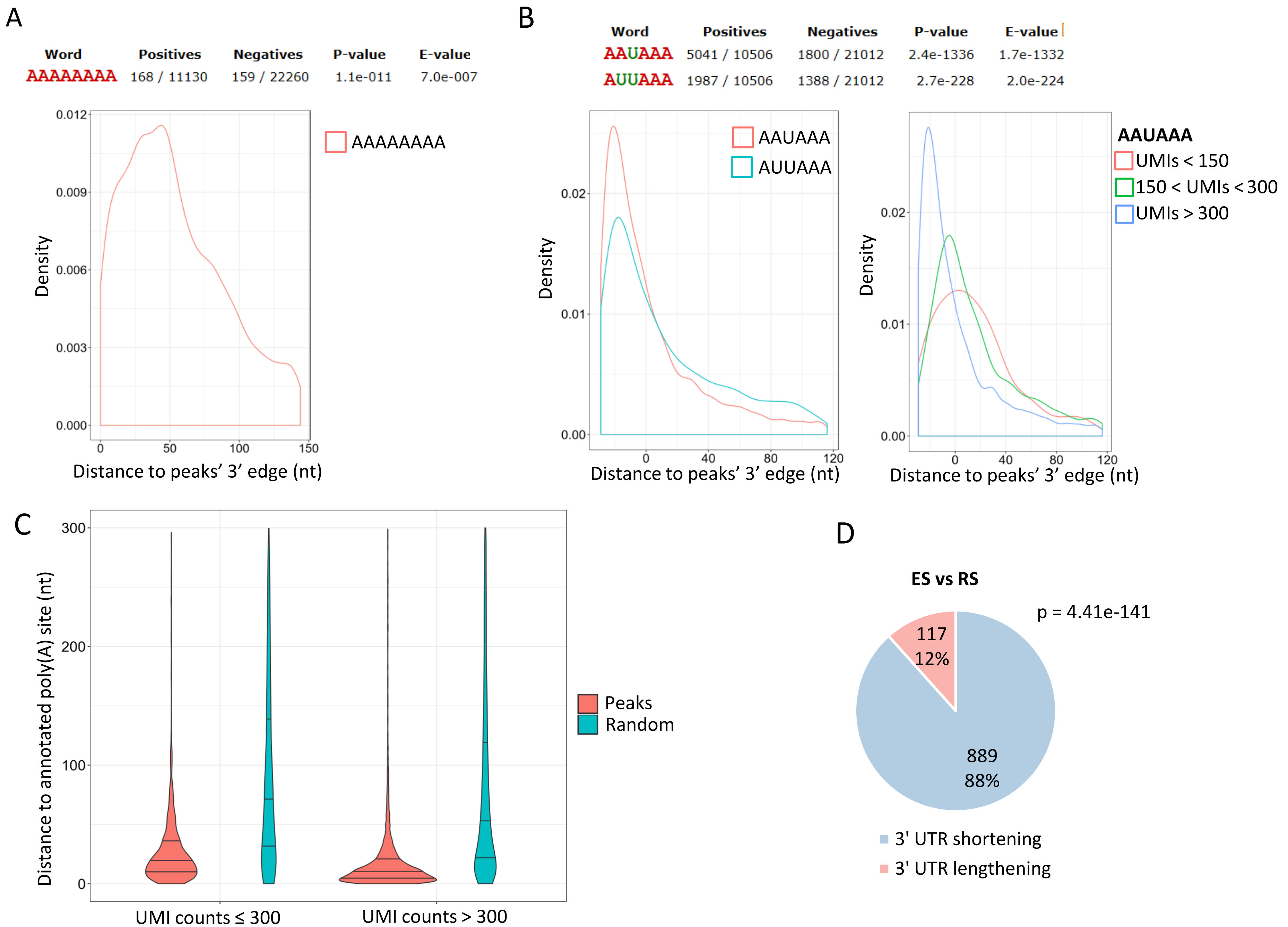
A
Word Positives Negatives P-value E-value
AAAAAAAA 168 / 11130 159 / 22260 1.1e-011 7.0e-007
AAAAAAAA
Density
Distance to peaks' 3' edge (nt)
B
Word Positives Negatives P-value E-value
AAUAAA 5041 / 10506 1800 / 21012 2.4e-1336 1.7e-1332
AUUAAA 1987 / 10506 1388 / 21012 2.7e-228 2.0e-224
AAUAAA
AUUAAA
AAUAAA
UMIs < 150
150 < UMIs < 300
UMIs > 300
Density
Distance to peaks' 3' edge (nt)
C
Distance to annotated poly(A) site (nt)
Peaks
Random
UMI counts ≤ 300
UMI counts > 300
D
ES vs RS
p = 4.41e-141
117
12%
889
88%
3' UTR shortening
3' UTR lengthening

Figure S3

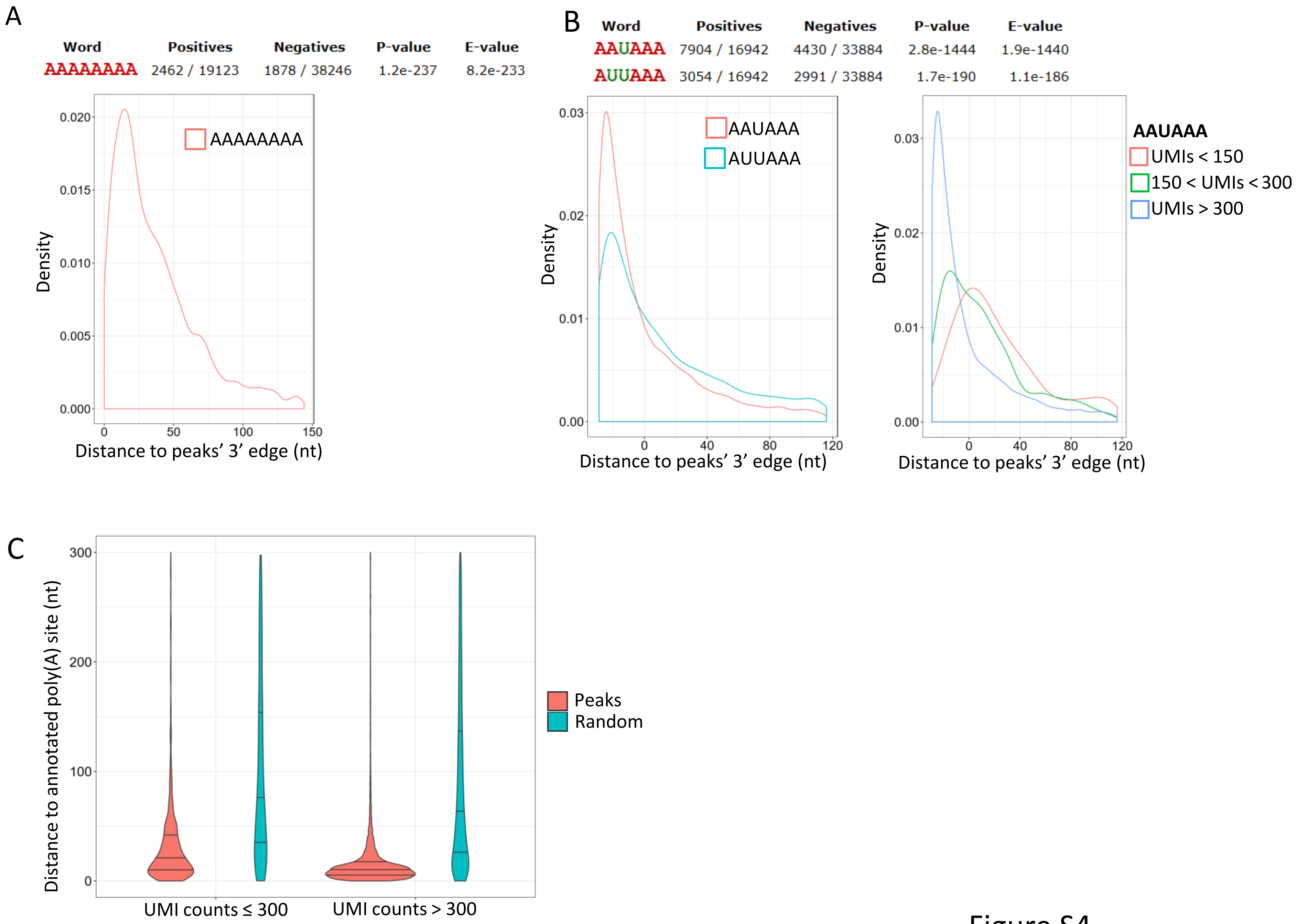

A
Word
Positives
Negatives
P-value
E-value
AAAAAAAA
2462 / 19123
1878 / 38246
1.2e-237
8.2e-233
AAAAAAAA
Density
0.020
0.015
0.010
0.005
0.000
0
50
100
150
Distance to peaks' 3' edge (nt)
B
Word
Positives
Negatives
P-value
E-value
AAUAAA
7904 / 16942
4430 / 33884
2.8e-1444
1.9e-1440
AUUAAA
3054 / 16942
2991 / 33884
1.7e-190
1.1e-186
AAUAAA
AUUAAA
Density
0.03
0.02
0.01
0.00
0
40
80
120
Distance to peaks' 3' edge (nt)
AAUAAA
UMIs < 150
150 < UMIs < 300
UMIs > 300
Density
0.03
0.02
0.01
0.00
0
40
80
120
Distance to peaks' 3' edge (nt)
C
Distance to annotated poly(A) site (nt)
300
200
100
0
UMI counts ≤ 300
UMI counts > 300
Peaks
Random

Figure S4

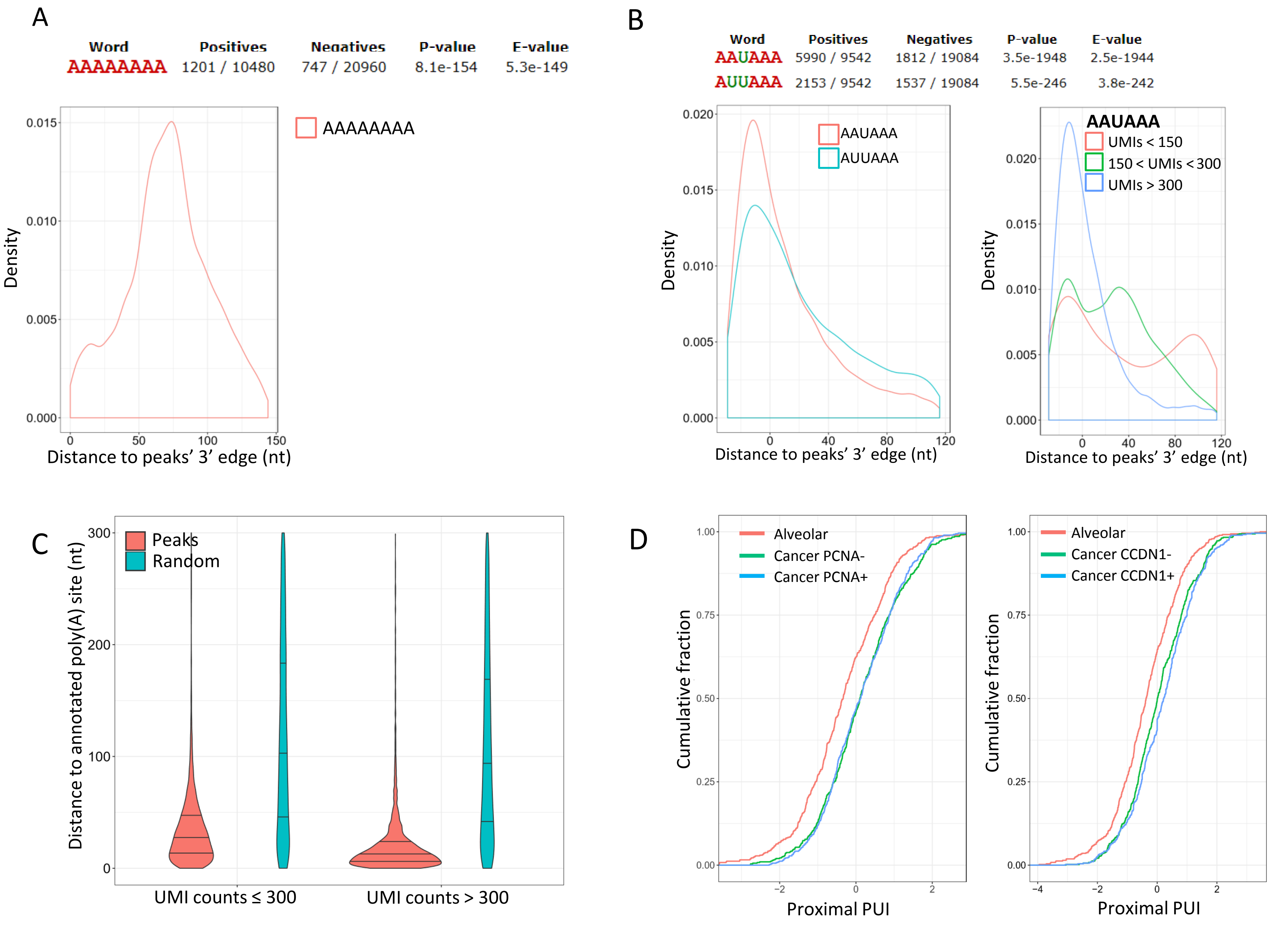
A
Word Positives Negatives P-value E-value
AAAAAAAA 1201 / 10480 747 / 20960 8.1e-154 5.3e-149
Density
Distance to peaks' 3' edge (nt)
B
Word Positives Negatives P-value E-value
AAUAAA 5990 / 9542 1812 / 19084 3.5e-1948 2.5e-1944
AUUAAA 2153 / 9542 1537 / 19084 5.5e-246 3.8e-242
AAUAAA
UMIs < 150
150 < UMIs < 300
UMIs > 300
C
Peaks
Random
Distance to annotated poly(A) site (nt)
UMI counts ≤ 300
UMI counts > 300
D
Alveolar
Cancer PCNA-
Cancer PCNA+
Cancer CCDN1-
Cancer CCDN1+
Cumulative fraction
Proximal PUI

Figure S5

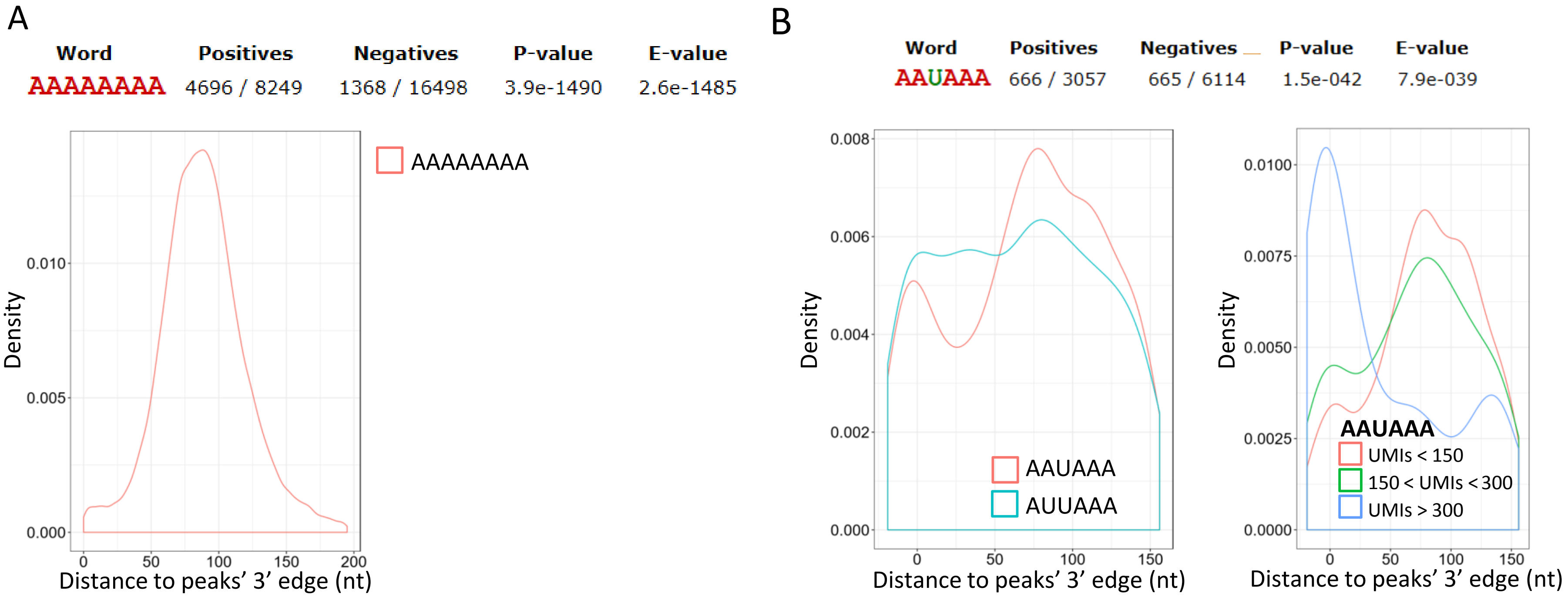
A
Word
Positives
Negatives
P-value
E-value
AAAAAAAA
4696 / 8249
1368 / 16498
3.9e-1490
2.6e-1485
Density
Distance to peaks’ 3’ edge (nt)
AAAAAAAA
B
Word
Positives
Negatives
P-value
E-value
AAUAAA
666 / 3057
665 / 6114
1.5e-042
7.9e-039
Density
Distance to peaks’ 3’ edge (nt)
AAUAAA
AUUAAA
Density
Distance to peaks’ 3’ edge (nt)
AAUAAA
UMIs < 150
150 < UMIs < 300
UMIs > 300

Figure S6

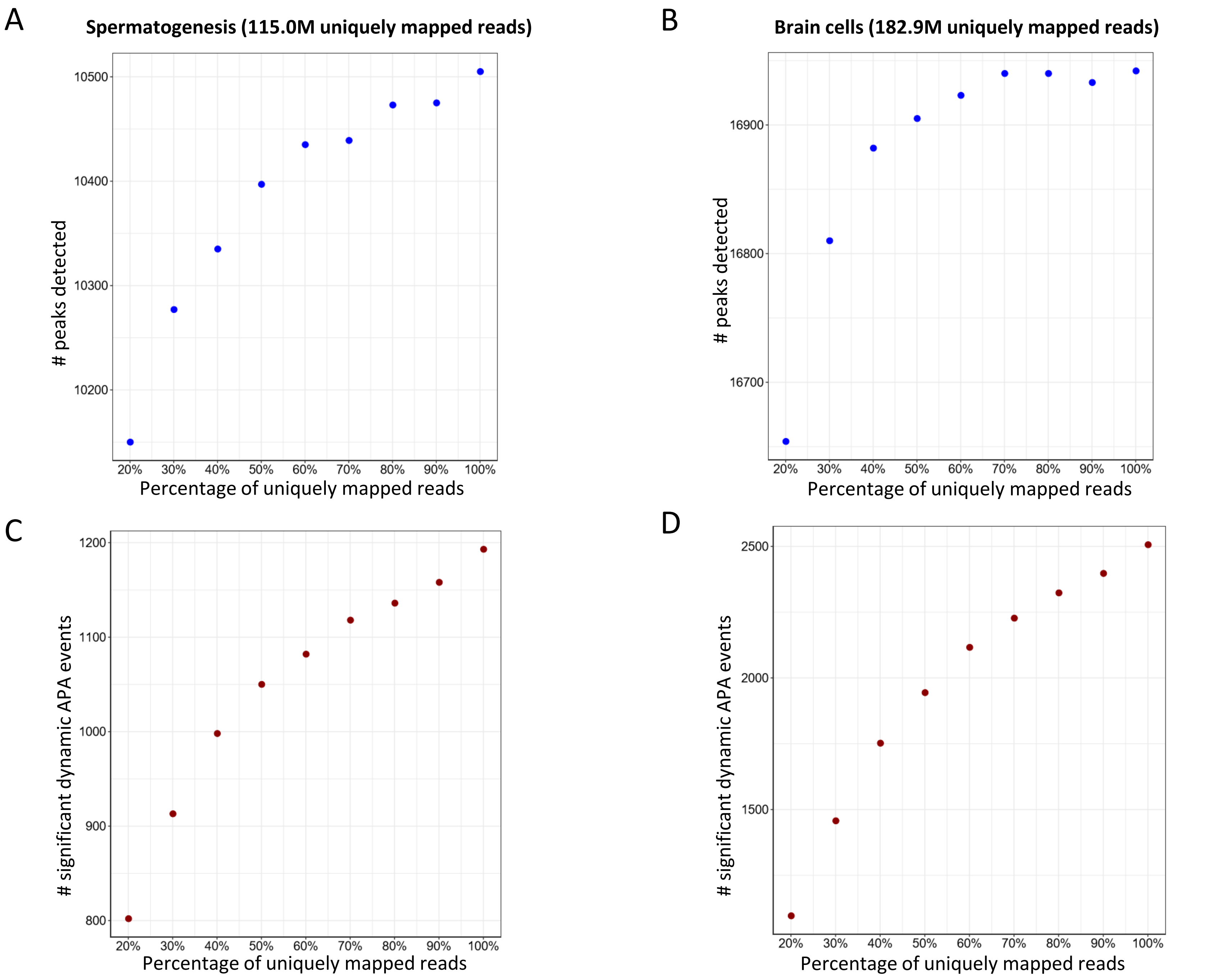
A
Spermatogenesis (115.0M uniquely mapped reads)
peaks detected
10500
10400
10300
10200
20%
30%
40%
50%
60%
70%
80%
90%
100%
Percentage of uniquely mapped reads
B
Brain cells (182.9M uniquely mapped reads)
peaks detected
16900
16800
16700
20%
30%
40%
50%
60%
70%
80%
90%
100%
Percentage of uniquely mapped reads
C
significant dynamic APA events
1200
1100
1000
900
800
20%
30%
40%
50%
60%
70%
80%
90%
100%
Percentage of uniquely mapped reads
D
significant dynamic APA events
2500
2000
1500
20%
30%
40%
50%
60%
70%
80%
90%
100%
Percentage of uniquely mapped reads

Figure S7

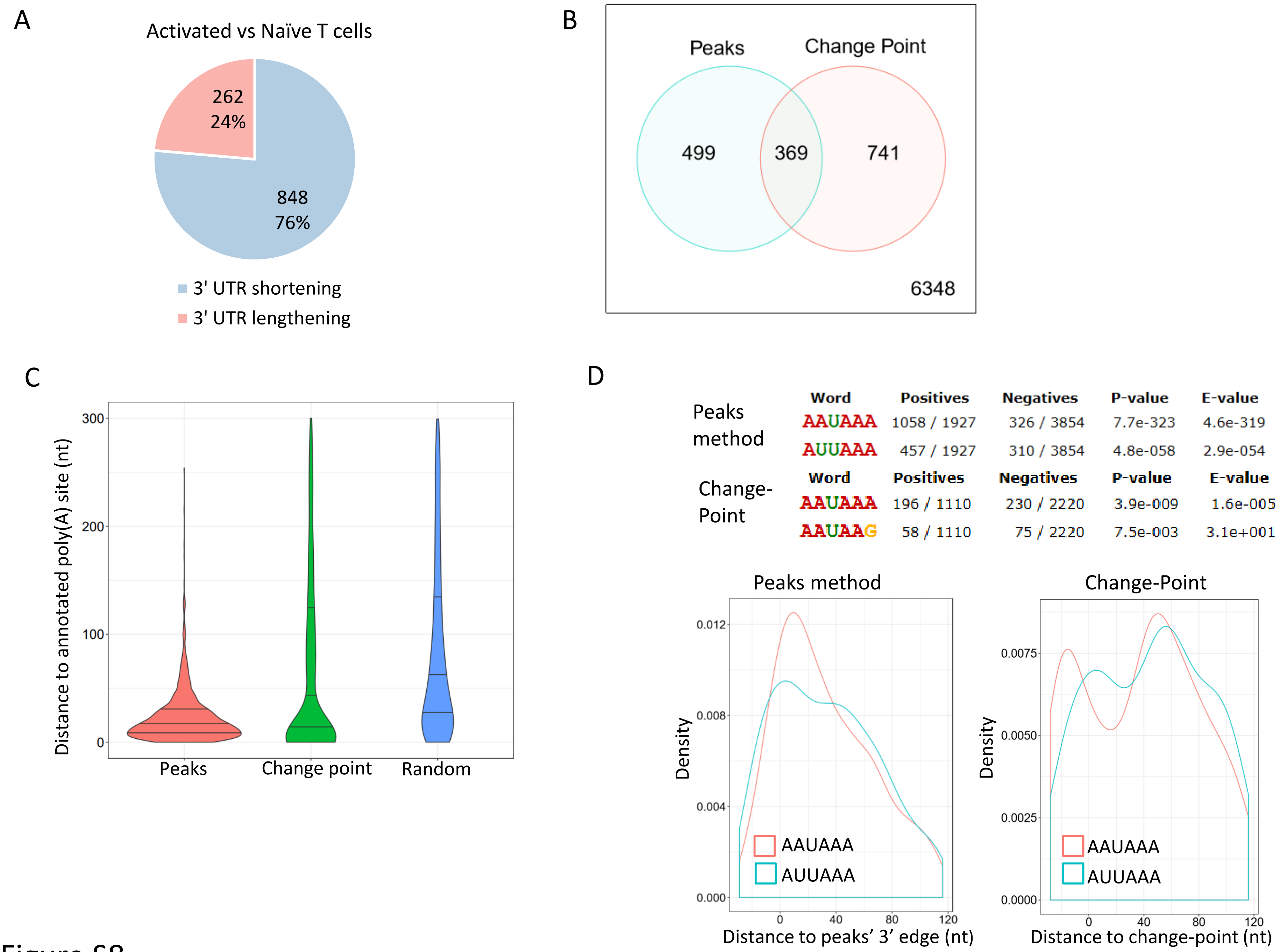
A
Activated vs Naïve T cells
262
24%
848
76%
3' UTR shortening
3' UTR lengthening
B
Peaks
Change Point
499
369
741
6348
C
Distance to annotated poly(A) site (nt)
300
200
100
0
Peaks
Change point
Random
D
Peaks method
Word | Positives | Negatives | P-value | E-value
AAUAAA | 1058 / 1927 | 326 / 3854 | 7.7e-323 | 4.6e-319
AUUAAA | 457 / 1927 | 310 / 3854 | 4.8e-058 | 2.9e-054
Change-Point
Word | Positives | Negatives | P-value | E-value
AAUAAA | 196 / 1110 | 230 / 2220 | 3.9e-009 | 1.6e-005
AAUAAG | 58 / 1110 | 75 / 2220 | 7.5e-003 | 3.1e+001
Peaks method
Density
0.012
0.008
0.004
0.000
0
40
80
120
AAUAAA
AUUAAA
Distance to peaks' 3' edge (nt)
Change-Point
Density
0.0075
0.0050
0.0025
0.0000
0
40
80
120
AAUAAA
AUUAAA
Distance to change-point (nt)

Figure S8

E

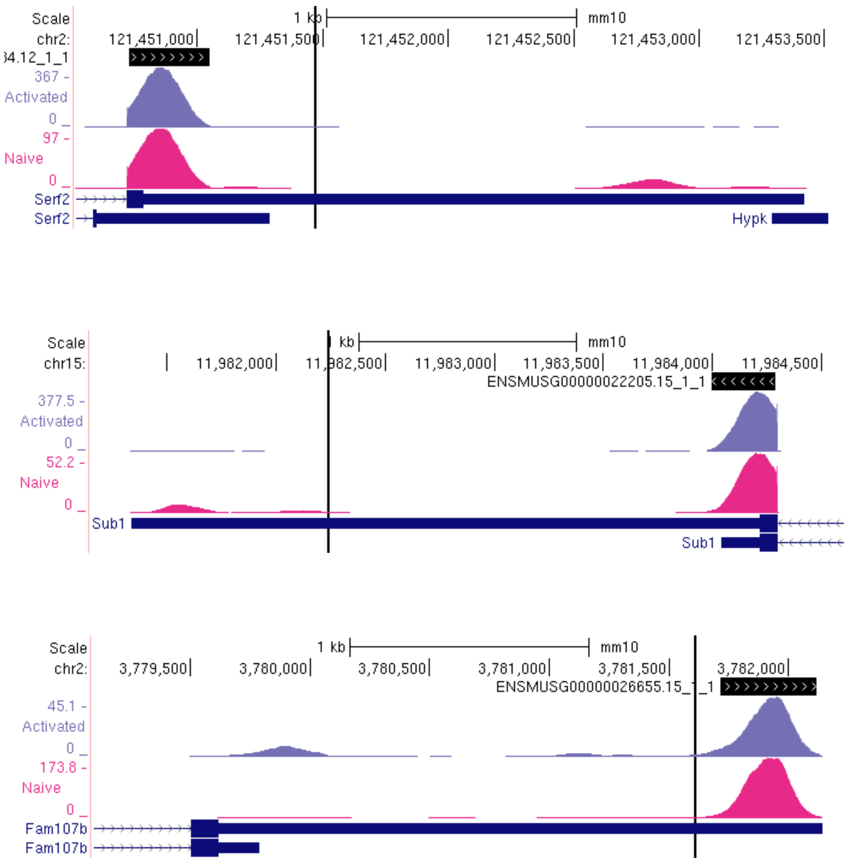

APA switch events detected by Change-Point but not by the Peak approach

F

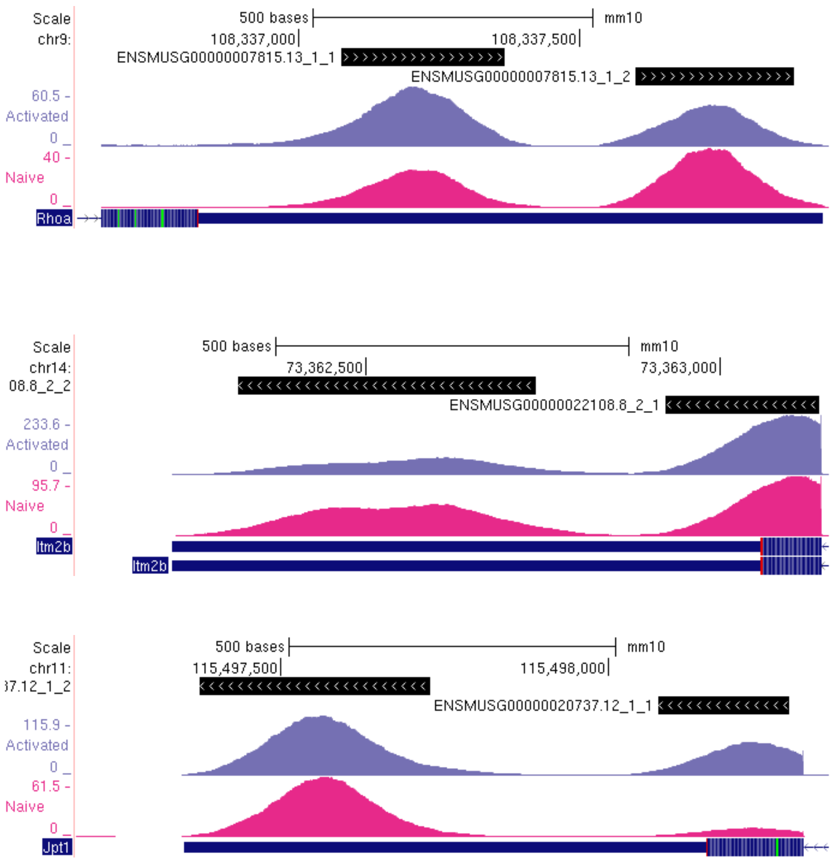

APA switch events detected by the Peak approach but not by Change-Point

Figure S8

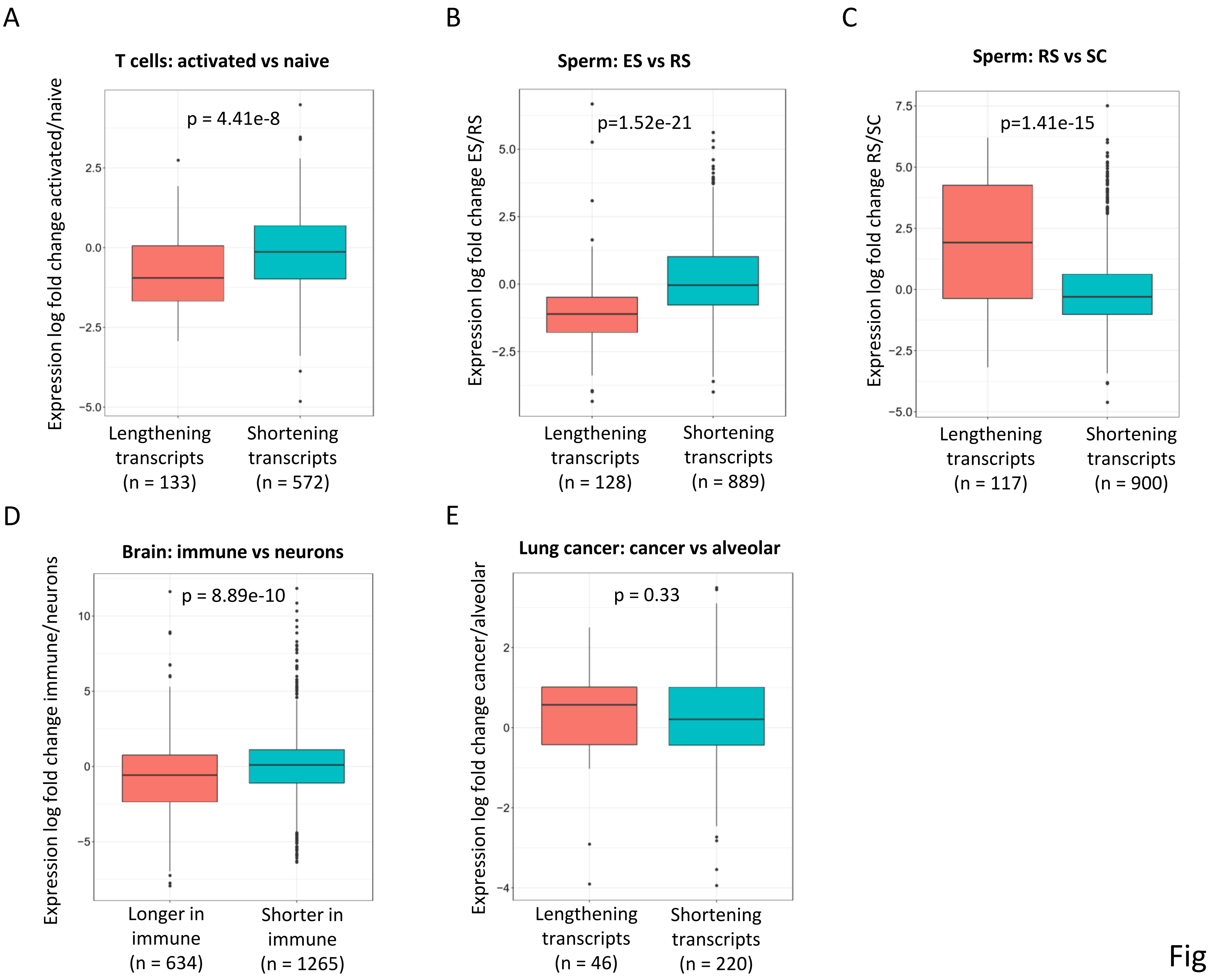
A
T cells: activated vs naive
p = 4.41e-8
Expression log fold change activated/naive
2.5
0.0
−2.5
−5.0
Lengthening transcripts (n = 133)
Shortening transcripts (n = 572)
B
Sperm: ES vs RS
p=1.52e-21
Expression log fold change ES/RS
5.0
2.5
0.0
−2.5
Lengthening transcripts (n = 128)
Shortening transcripts (n = 889)
C
Sperm: RS vs SC
p=1.41e-15
Expression log fold change RS/SC
7.5
5.0
2.5
0.0
−2.5
−5.0
Lengthening transcripts (n = 117)
Shortening transcripts (n = 900)
D
Brain: immune vs neurons
p = 8.89e-10
Expression log fold change immune/neurons
10
5
0
−5
Longer in immune (n = 634)
Shorter in immune (n = 1265)
E
Lung cancer: cancer vs alveolar
p = 0.33
Expression log fold change cancer/alveolar
2
0
−2
−4
Lengthening transcripts (n = 46)
Shortening transcripts (n = 220)

Figure S9

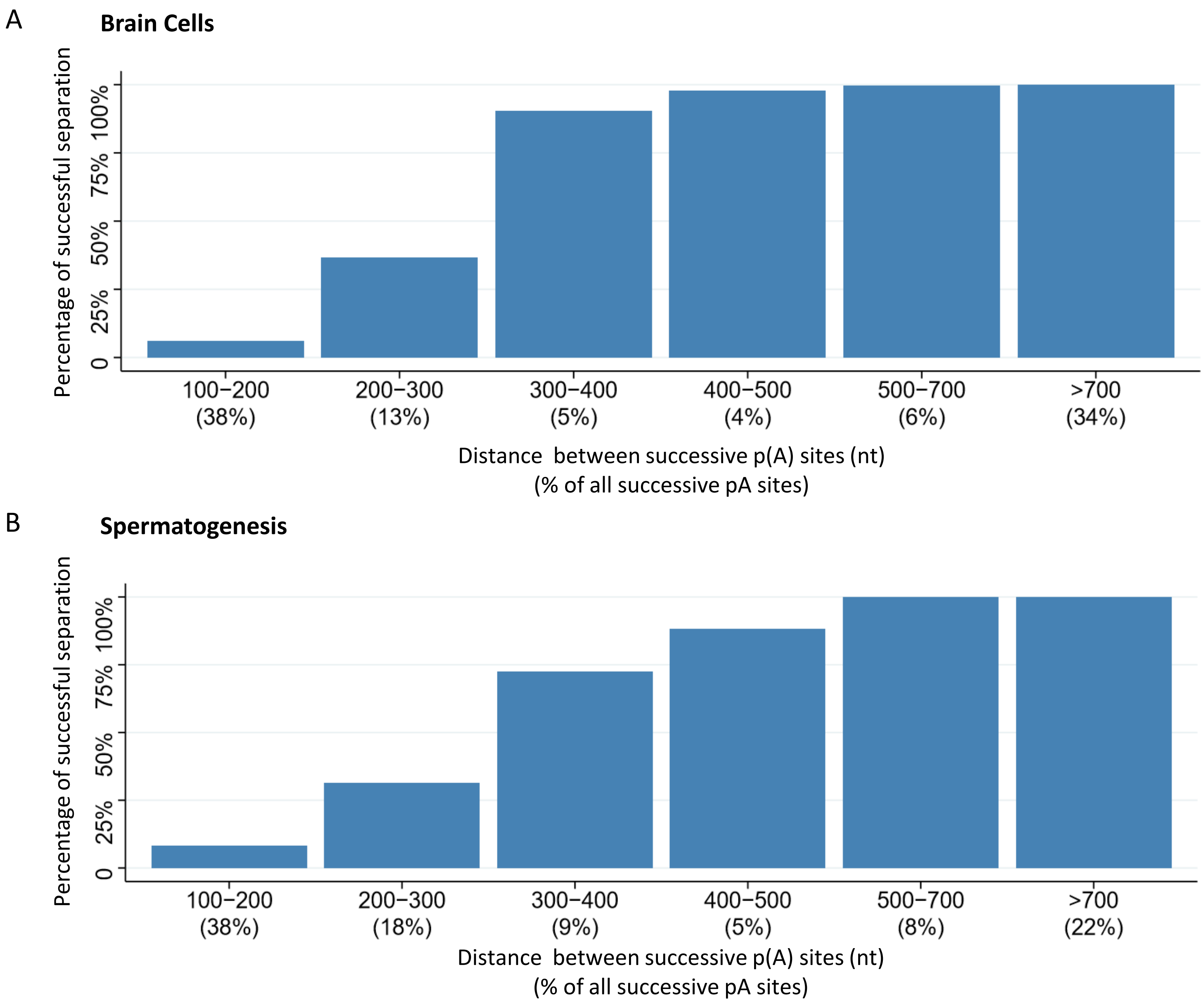
A
Brain Cells
Percentage of successful separation
0
25%
50%
75%
100%
100−200 (38%)
200−300 (13%)
300−400 (5%)
400−500 (4%)
500−700 (6%)
>700 (34%)
Distance between successive p(A) sites (nt)
(% of all successive pA sites)
B
Spermatogenesis
Percentage of successful separation
0
25%
50%
75%
100%
100−200 (38%)
200−300 (18%)
300−400 (9%)
400−500 (5%)
500−700 (8%)
>700 (22%)
Distance between successive p(A) sites (nt)
(% of all successive pA sites)

Figure S10
